# Fast Screening of Inhibitor Binding/Unbinding Using Novel Software Tool CaverDock

**DOI:** 10.3389/fchem.2019.00709

**Published:** 2019-10-29

**Authors:** Gaspar P. Pinto, Ondrej Vavra, Jiri Filipovic, Jan Stourac, David Bednar, Jiri Damborsky

**Affiliations:** ^1^Loschmidt Laboratories, Department of Experimental Biology and RECETOX, Faculty of Science, Masaryk University, Brno, Czechia; ^2^International Centre for Clinical Research, St. Anne's University Hospital Brno, Brno, Czechia; ^3^Institute of Computer Science, Masaryk University, Brno, Czechia

**Keywords:** binding, docking, channel, unbinding, virtual screening, inhibitors, substrates, tunnel

## Abstract

Protein tunnels and channels are attractive targets for drug design. Drug molecules that block the access of substrates or release of products can be efficient modulators of biological activity. Here, we demonstrate the applicability of a newly developed software tool CaverDock for screening databases of drugs against pharmacologically relevant targets. First, we evaluated the effect of rigid and flexible side chains on sets of substrates and inhibitors of seven different proteins. In order to assess the accuracy of our software, we compared the results obtained from CaverDock calculation with experimental data previously collected with heat shock protein 90α. Finally, we tested the virtual screening capabilities of CaverDock with a set of oncological and anti-inflammatory FDA-approved drugs with two molecular targets—cytochrome P450 17A1 and leukotriene A4 hydrolase/aminopeptidase. Calculation of rigid trajectories using four processors took on average 53 min per molecule with 90% successfully calculated cases. The screening identified functional tunnels based on the profile of potential energies of binding and unbinding trajectories. We concluded that CaverDock is a sufficiently fast, robust, and accurate tool for screening binding/unbinding processes of pharmacologically important targets with buried functional sites. The standalone version of CaverDock is available freely at https://loschmidt.chemi.muni.cz/caverdock/ and the web version at https://loschmidt.chemi.muni.cz/caverweb/.

## Introduction

Until the beginning of the new millennium, drug design mostly relied on experimental high-throughput screening (Kansy et al., 1998; Zhang et al., 1999; Bleicher et al., 2003). These techniques evolved rapidly up to the beginning of the nineties. However, although at that time they seemed promising and the best techniques for drug design and discovery, they were expensive in both time and labor (Bajorath, 2002; Bielska et al., 2014). More cost-effective methods emerged with the introduction of docking algorithms and thorough analysis of protein-ligand interactions. This boom in docking approaches led to the development of over 60 software tools for docking (Sousa et al., 2010; Pagadala et al., 2017). At the beginning of the new millennium, a new technique for drug design called “virtual screening” started to gain recognition (Clark, 2008;Ripphausen et al., 2010).

Virtual screening is now a well-established technique for drug design (Bottegoni et al., 2016), both in academic research and the pharmaceutical industry (Mangoni et al., 1999; Clark, 2008; Huang et al., 2008; Totrov and Abagyan, 2008; Cheng et al., 2012; Kaczor et al., 2016). Many docking programs are available for virtual screening and several comparisons and benchmarks have been published (Cummings et al., 2005; Cross et al., 2009; Lavecchia and Di Giovanni, 2013; Bielska et al., 2014; Chaput et al., 2016; Kim et al., 2016). These programs help in the first step of the drug design process and follow a general protocol of screening a large database of small compounds on a chosen target (receptor). After selecting a target, a library of ligands is chosen. The ligands can be taken from many publically available or commercial libraries. Of these, ZINC (http://zinc15.docking.org/) (Sterling and Irwin, 2015), ChEMBL (https://www.ebi.ac.uk/chembl/) and PubChem (https://pubchem.ncbi.nlm.nih.gov/) are among the most widely used and largest ligand libraries. However, other databases with fewer compounds may be useful when searching for compounds with specific characteristics. An example is Drugbank (https://www.drugbank.ca/) (Law et al., 2014), which is a database of drugs approved by the FDA and Canadian Agency for Drugs and Technologies in Health. It also enables selection of experimental, investigational and illicit drugs.

The success of virtual screening boosted the development of techniques used for drug design and in recent years, binding kinetics has gained increased momentum in the drug design community. A research program supported by the European Innovative Medicine Initiative has, for the last 6 years, focused on understanding target binding kinetics (Laverty et al., 2012; Goldman et al., 2013; Kush and Goldman, 2014). Although there has been a steep rise in the development of methods for drug design, there is still space for further improvements.

The binding of a substrate and release of the products of an enzymatic reaction have been studied using different computational approaches (Straatsma and McCammon, 1992; Kollman, 1993; Lamb and Jorgensen, 1997). Classical and accelerated molecular dynamics simulations have been used to calculate ligand binding affinities. These methods use the free energy perturbation approach to calculate the relative binding free energy between a receptor and two ligands based on the thermodynamic cycle (Kruse et al., 2012; Tomić et al., 2015). However, such methods are computationally demanding and not suitable for screening large libraries. Development of new approaches for analysis of ligand binding and unbinding is clearly needed.

Several computational tools have been developed for searching the best binding positions in the active site pocket of a target molecule and then binding positions with increasing distance from the active site. PELE is a web server that incorporates a wide range of different types of calculation, including protein local motions (Lucas and Guallar, 2012). PELE also enables ligand binding refinement, binding site searches and ligand migration. The latter three scripts yield multiple-pose docking results through all protein free space, which cannot be achieved with simple docking algorithms (Guallar et al., 2009; Hernández-Ortega et al., 2011; Espona-Fiedler et al., 2012; Madadkar-Sobhani and Guallar, 2013). MOMA-LigPath (Devaurs et al., 2013) has a robotics algorithm for space search, not only in the active site pocket but also along an unbinding trajectory. However, as the tool does not output information on the energy of conformations, it is not possible to prioritize individual pathways. SLITHER (Lee et al., 2009) is a web server built to generate conformations of substrates while traveling through membrane channels. It is based on both the AUTODOCK (Morris et al., 2009) and MEDock (Chang et al., 2005) docking algorithms. Energetic information is available from these calculations. However, docking trajectories are often sparse.

We have developed a fast method based on analysis of protein tunnels (Marques et al., 2017) combined with molecular docking in a single implementation—called CaverDock—and used it to address important biochemical problems. Protein tunnels are structural features connecting the buried active site cavities with the protein surface. First, tunnels in proteins are identified using the specialized software Caver (Chovancova et al., 2012). Then, an extensively optimised version of AutoDock Vina (Trott and Olson, 2010) is used to dock a ligand along the tunnel to produce a continuous trajectory. Algorithms implemented in CaverDock (Filipovic et al., 2019; Vavra et al., 2019) can be used to run a virtual screening protocol for binding a library of ligands into and from the active site. This procedure identifies energetically favorable binding sites located outside the active site, providing a profile of potential energies. The goal of CaverDock, in current implementation, is not the calculation of the free energy of binding. Instead of obtaining several trajectories to calculate the free energy (Jarzynski, 1997; Fernández, 2014), CaverDock calculates the binding energy along the several, predetermined, points along the tunnel.

We have utilized the new CaverDock tool in three applications. The first examined differences between substrates and inhibitors and selection of flexible side chains along tunnels bottlenecks, which serve as potential hot spots for mutagenesis. The datasets used for testing of the flexible simulations consisted of seven proteins with six tunnels and one channel: (i) cytochrome P450 17A1, (ii) leukotriene A4 hydrolase/aminopeptidase, (iii) acetylcholinesterase (AChE), (iv) human plasma cholesteryl ester transfer protein (CETP), (v) inducible nitric oxide synthase (iNOS), (vi) UDP-3-O-N-acetylglucosamine deacetylase (LpxC), and (vii) matrix metalloproteinase-13 (MMP-13). Trajectories were calculated for both the natural substrates and inhibitors. The second application was the study of human N-terminal domain of heat shock protein 90α (N-HSP90), an important pharmaceutical cancer target, with a diverse set of inhibitors. The dataset was obtained from previously published study (Kokh et al., 2018). We compared the resulting conformations from CaverDock with positions of inhibitor molecules found in the crystal structures. Furthermore, we analyzed the correlations between CaverDock energies and measured experimental values (Kokh et al., 2018). The third application was the screening of potential inhibitors and identification of the access pathways through simulation of binding processes. The applicability of CaverDock for virtual screening pharmaceutically important molecules was validated with cytochrome P450 17A1 and a dataset of oncological drugs from the NIH.gov website and with leukotriene A4 hydrolase/aminopeptidase and a dataset of anti-inflammatory drugs from the drugbank.ca website. The presented results demonstrate that CaverDock is a ready-to-use tool that should be of broad interest to biochemists, protein engineers, and medicinal chemists.

## Methods

### Protein Targets

Cytochrome P450 17A1 and leukotriene A4 hydrolase/aminopeptidase were selected for flexibility testing as well as the model systems to validate the applicability of CaverDock for the virtual screening of ligand libraries. Seven protein targets were considered, as described below. The description of the structure and function of Acetylcholinesterase (AChE), Cholesteryl ester transfer protein (CETP), Nitric oxide synthase (iNOS), Metal-dependent deacetylase (LpxC), and Matrix metalloproteinase-13 (MMP-13) is provided in the Supplementary Information.

*Cytochrome P450 17A1* functions as a drug-processing enzyme and was selected as the target protein for both application studies. The starting structure for this work was the crystal structure taken from the Protein Data Bank (Berman et al., 2000) with PDB-ID 3RUK (DeVore and Scott, 2012). The structure comprised an agglomerate of 4 cytochromes P450 17A1, from which we only used chain A. The structure also contained the inhibitor Abiraterone, which blocked access to the active site and was deleted prior to CaverDock screening.

*Leukotriene A4 hydrolase/aminopeptidase*, with crystal structure PDB-ID 4L2L (Stsiapanava et al., 2014), was selected as the second target for both application studies. Leukotrienes are a family of lipid mediators that play important roles in a variety of allergic and inflammatory reactions (Haeggström et al., 1990; Funk, 2001; Haeggström, 2004; Szul et al., 2016). Leukotriene A4 hydrolase/aminopeptidase (EC 3.3.2.6) is a bifunctional zinc metalloenzyme that catalyzes formation of the chemotactic agent LTB4, a key lipid mediator in the immune response. This enzyme, had an inhibitor, 4-(4-benzylphenyl) thiazol-2-amine, bound to the crystal structure, which had to be removed prior to screening. LTA4H possesses two known activities, both of which are exerted via distinct but overlapping active sites and depend on a catalytic zinc atom. The catalytic zinc atom is bound to the signature HEXXH, known also for other M1 metallopeptidases (Gomis-Rüth et al., 2012; Zhang et al., 2015).

*Heat shock protein 90*α (HSP90) is a chaperone protein that assists the folding of client proteins. The HSP90 consists of three domains. The highly conserved N-terminal domain with ATP-binding cleft which is responsible for the catalytic activity. The middle domain contains a large hydrophobic surface needed for the folding of client proteins. The C-terminal domain is involved in the dimerization of HSP90 (Li et al., 2012). The function of HSP90 is linked to hydrolysis of ATP and the dimerization. A number of the HSP90 client proteins are part of cancer cell-associated signaling pathways, therefore the HSP90 is an important target in drug design. The function of HSP90 can be blocked by small molecules. This inhibition leads to degradation of the client proteins and impacts tumor growth (Kabakov et al., 2010). In this study, we analyzed the bound (HOLO) crystal structures with several small inhibitors inside the ATP-binding pocket. Furthermore, we conducted CaverDock simulations with a larger set of inhibitors using the unbound (APO) crystal structure of N-HSP90 (Kokh et al., 2018).

### Structural Analysis of N-HSP90 HOLO Complexes

We studied the ability of CaverDock to find protein-ligand conformations similar to the crystal structures using the set of previously published complexes (Kokh et al., 2018). We analyzed the 34 crystal complexes of the N-HSP90 with different co-crystallized inhibitors. The list of the PDB IDs is in Supplementary Table S1. The crystal structures were aligned by DeepAlign (Wang et al., 2013) to simplify the following analyses. The tunnels for CaverDock runs were calculated by Caver 3.02 (Chovancova et al., 2012) in each inhibitor-free structure starting from the catalytic residues 93 and 138 with the probe radius, shell radius and shell depth set to 1.5, 20, and 20 Å, respectively. The tunnel leading through the main opening of the ATP-binding cleft to the active site was selected, discretized with 0.3 Å steps and extended by 20 Å to ensure complete unbinding of the tested inhibitor molecules. The receptor and ligand PDBQT files for CaverDock were prepared by MGLtools (Morris et al., 2009).

### Energy Analysis of N-HSP90 HOLO and APO Complexes

Based on the previously published kinetic data (Kokh et al., 2018), we prepared two datasets. The first dataset consists of a subset of 32 inhibitors and HOLO structures from the HOLO structure analysis dataset described above. The kinetic data for two inhibitors (compound_01 and compound_04) was not complete in the original publication. The second dataset was created to check the findings from the HOLO dataset. It consists of 68 inhibitors. In this case, we ran the CaverDock calculations with the APO structure of N-HSP90 (PDB ID 3T0H). The CaverDock calculations were carried out in the same manner as described above for the structural analysis.

### Libraries of Small Ligands

Several approaches can be used to choose libraries for virtual screening. For instance, screening as many ligands as possible from a broad dataset of molecules, such as the ZINC database. Another approach is to screen for drug-like compounds with specific biological activities. Virtual screening may also be performed using cognate ligands belonging to a group of compounds that the enzyme naturally binds and/or catalyzes. In the present study, we conducted a virtual screening campaign on a group of drug-like molecules possessing predefined biological activities. The chosen ligands were converted to the AutoDock Vina-compatible PDBQT format using MGLTools v1-5-7rc1 (Morris et al., 2009). We used the inhibitors complexed in the structures for validation of flexible side chains (inhibitor dataset). We built the substrates in Avogadro and minimized them with the UFF forcefield (Hanwell et al., 2012) for the natural substrates (substrate dataset).

A dataset of *133 cancer drugs* was downloaded from the NIH.gov website for the cytochrome P450 17A1. The drugs were all FDA-approved and have been used against different types of cancer. Of the 133 drugs, 105 were used for the screening and 28 were excluded due to being salts or having unconventional atoms that could not be properly handled by AutoDock Vina. Among the 28 excluded drugs, 22 had two ligand molecules in the same file. The other six drugs had some atoms (one with arsenic, three with platinum and two with boron) for which there were no parameters available in the force field of AutoDock Vina. A dataset of *56 anti-inflammatory drugs* was downloaded from the drugbank.ca website for leukotriene A4 hydrolase/aminopeptidase. Out of these 56 drugs, 54 were used and 2 were excluded from the screening. One excluded drug contained a gold atom, for which AutoDock Vina had no defined parameters. The other drug was a silicate mineral with two molecules in the same file.

### CaverDock Calculation

**Figure 1:**
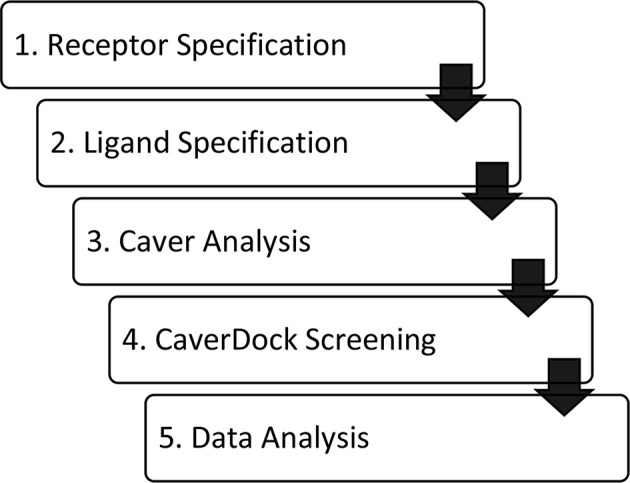
Workflow of virtual screening using CaverDock. (1) Receptor and (2) ligand specification follow established protocols of the software tool AutoDock (Morris et al., 2009). (3) Identification of protein tunnels using Caver (Chovancova et al., 2012). (4) Tunnel discretization and sequential ligand binding study using CaverDock (Filipovic et al., 2019; Vavra et al., 2019). (5) Analysis of docking trajectories and energy profiles, extraction of energy barriers and protein-ligand complexes possessing the lowest energies.

The software tool CaverDock is available free of charge at the website https://loschmidt.chemi.muni.cz/caverdock/. The CaverDock protocol (Figure 1) starts with finding the tunnels by using Caver (Chovancova et al., 2012). Caver can be used as a standalone program or as a Pymol plugin. The active site is selected as a starting point for the Caver calculation. For all seven proteins, the location of ligand binding in the active site was known (Gerber et al., 1997; Funk, 2001; Thunnissen et al., 2001; Epps and Vosters, 2002; Rudberg et al., 2002; Haeggström et al., 2007; Gattis et al., 2010; Singh and Konwar, 2012; Clayton et al., 2013; Khatri et al., 2014; Yoshimoto and Auchus, 2015). All the other settings were fixed at default values. This step of the protocol yields several tunnels for each protein. The numbering of the tunnels is given by a parameter called priority, which is the ratio between (i) bottleneck radius, (ii) tunnel length and (iii) curvature of the tunnel. The tunnels were represented as a series of sequential spheres.

We only used tunnels with priority 1 for the flexible simulations. These tunnels had the inhibitor already inside, although we removed it before the Caver calculation since we did not relax the protein in any way. Hence, there was an implicit bias for these tunnels. The flexibility in CaverDock arises from the already implemented flexibility capabilities of Autodock Vina. Flexibility on side chains was introduced in three iterations. In the default mode, CaverDock adds flexibility to two residues in each iteration, up to three iterations. These values may be changed by the user to better fit their needs. For each tunnel in the substrate and inhibitor datasets, two flexible bottleneck residues were added in each iteration. These flexible residues were not necessarily the same for the substrate and inhibitor simulations. Since substrates and inhibitors may differ in length and volume, the bottlenecks that they encounter along a tunnel may also differ.

For cytochrome P450 17A1, we used three tunnels for our virtual screening study. The first two tunnels found by Caver were also described in the literature, whereas the third tunnel was ranked as #5 by Caver. By individually inspecting every tunnel, we noted that tunnels ranked #3 and #4 by Caver were too long and narrow to be feasible as a ligand access pathway. For leukotriene A4 hydrolase/aminopeptidase, we used two tunnels ranked #1 and #2 by Caver. The results obtained were consistent with the literature (Cui et al., 2015), confirming that these two tunnels were used by the protein to transport ligands/drugs in and products out. Since the active site in leukotriene A4 hydrolase/aminopeptidase is inserted deeper into the protein and the protein itself is packed closer together than in cytochrome P450 17A1, only six tunnels were described for this protein vs. 15 tunnels described for cytochrome P450 17A1. A literature search showed that the tunnels ranked highest by Caver were indeed tunnels used by the natural substrate and inhibitors (Yu et al., 2013; Stsiapanava et al., 2014).

**Figure 2:**
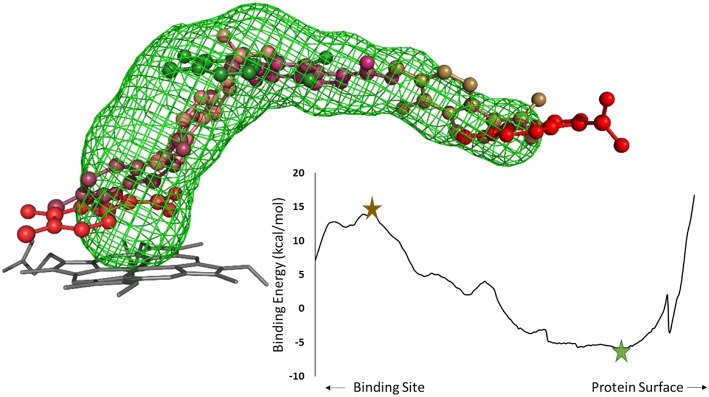
Analysis of an inhibitor passing through a tunnel using CaverDock. Left: structure of cytochrome P450 17A1 and tunnel #3 with seven (7/254) snapshots of a ligand bound along the tunnel. The minimum binding energy conformation is shown in green, whereas the maximum binding energy is shown in brown, in a ball-and-stick representation. Right: energy profile obtained from the CaverDock calculation for cytochrome P450 17A1 and its tunnel #3 with the ligand.

After selecting the tunnels to study, the next step in a CaverDock protocol is to discretize the tunnels. Tunnel discretization divides each tunnel into a set of discs. The ligand is *glued* to a disc by one of its atoms and as the disc moves through the tunnel, the software defines a ligand path coordinate. After discretization, we extended the tunnels by two Ångströms. This step ensured that the tunnels were long enough to enable identification of the local binding minima at the tunnel mouth. Having prepared the tunnels, we used MGL tools to set the AutoDock atom types and Gasteiger charges for the receptor and ligands. MGL tools provide scripts that convert pdb and mol2 files into pdbqt file format. Having prepared the receptors and ligands, we next prepared a CaverDock file to run the docking step. This file was equivalent to the one used by AutoDock Vina but with the path to the file containing tunnel information instead of the receptor (Trott and Olson, 2010). We then added information about the studied tunnel from Caver to the configuration file. This new information allowed the “docking” conformation to be searched along the tunnel on each disc. One configuration file needs to be created for each ligand. Figure 2 shows a representation of a ligand bound along a tunnel taken from several snapshots of a CaverDock calculation.

An AutoDock Vina virtual screening was also performed with the same targets as the CaverDock virtual screening. To ensure that AutoDock Vina yielded the best result possible within a reasonable time, we used an exhaustiveness setting of 30. The center of the matrix grid was the same as that used for the CaverDock calculation and the box was 27 Å on each side.

## Results

### Simulations With Flexible Side Chains

**Figure 3:**
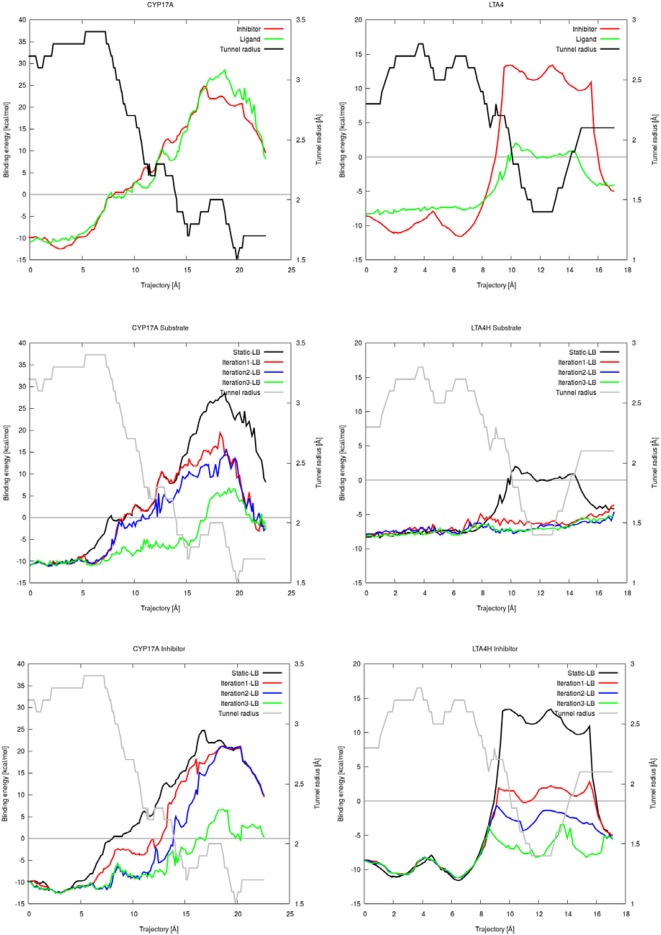
Plots of the binding energies of cytochrome P450 17A1 (left) and leukotriene A4 hydrolase/aminopeptidase (right) obtained from CaverDock with and without flexibility. Binding energies between substrate and inhibitor with tunnel radius present (top). Binding energies from substrate simulations with flexibility, rigid simulation, and tunnel radius on the background (middle). Binding energies from all inhibitor simulations with flexibility, rigid simulation and tunnel radius on the background (bottom).

CaverDock allows flexibility of residue side chains along a tunnel. We tested the intrinsic flexibility of AutoDock Vina implemented in CaverDock with the substrate and inhibitor datasets. We introduced flexibility in three iterations by adding two flexible bottleneck residues in each iteration. Thus, iteration 1, 2, and 3 had two, four and six flexible side chains, respectively. The energetic barrier for the substrate and inhibitor to travel from the inside to outside of cytochrome P450 17A1 or leukotriene A4 hydrolase/aminopeptidase was lowered when we added flexible side chains (Figure 3). For cytochrome P450 17A1, the binding energy was lowered with each iteration for both the inhibitor and substrate. In the case of the substrate of leukotriene A4 hydrolase/aminopeptidase, the energetic barrier was stabilized with only two flexible side chains and addition of further flexible side chains gave no apparent change in the energetic barrier along the trajectory of the substrate. On the other hand, the inhibitor of leukotriene A4 hydrolase/aminopeptidase had a lower energetic barrier with each iteration. As expected, the inhibitor had a similar or more stable binding energy when compared to the substrate.

We showed that the flexible simulations were able to open parts of the tunnel with high barriers with the substrate and inhibitor datasets (electronic SI). Significant energetic barriers were lost in the iteration with six flexible residues. In this scenario, the ligand was able to leave without any spatial or energetic hindrance. The flexible side chains moved out of the way to let the ligand escape, but the new conformations of side chains were close to the rest of the protein structure. Adding flexible residues did not affect the energetic barrier in iNOS, which showed a similar profile through all iterations in the ligand simulations (electronic SI). In these cases, the tunnel radius was already large enough for unrestricted ligand exchange with no obvious bottleneck.

The usage of the intrinsic AutoDock Vina flexibility in CaverDock is still under development and new algorithms are being tested for obtaining better results. With the current version, users are advised to use both rigid and flexible simulations with four or less flexible side chains. We can get more information about the tunnel with the flexible side chains, e.g., to identify which residues need to be flexible to open the tunnel for ligand passage since these residues are natural hot-spots for potential mutagenesis. However, there is an obvious computational price to pay when using flexible simulations, as shown in Table 1. In particular, adding flexible residues leads to longer simulation times. We advise running CaverDock simulations with lower bound trajectories only when running in a rigid mode because rigid trajectories may yield unrealistic high barriers when running upper bound simulations (discussed below).

**Table 1:** Summary of calculation times for lower bound calculations with rigid and flexible simulations and indication of the flexible residues added on each iteration.

		**Cytochrome P450 17A1**		**Leukotriene A4 hydrolase/aminopeptidase**	
		**Inhibitors**	**Substrates**	**Inhibitors**	**Substrates**
Rigid	Time	47 min 48 s	48 min 13 s	205 min 32 s	24 min 39 s
1st Iteration	Time	110 min 35 s	66 min 18 s	338 min 41 s	50 min 32 s
	Flexible Residues	Ile205, Tyr201	Ile205, Ile246	Val381, Val367	Val367, Phe362
2nd Iteration	Time	252 min 28 s	168 min 3 s	854 min 20 s	112 min 56 s
	Flexible Residues	Arg239, His235	Tyr201, His235	Lys364, Phe362	His360, Lys364
3rd Iteration	Time	436 min 20 s	332 min 22 s	1,994 min 1 s	156 min 11 s
	Flexible Residues	Ile198, Leu242	Leu243, Arg239	His360, Lys385	Gln136, Ile372

*Residues are chosen automatically to lower potential energy at bottlenecks*.

### Comparison of Calculated and Experimental Results

#### Structural Analysis of HOLO Structures

**Figure 4:**
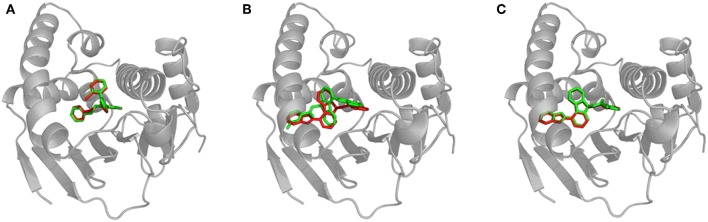
Structural comparison of CaverDock binding poses and X-ray structures. **(A)** Compound_14 correctly predicted by CaverDock (green) with low RMSD compared to the original crystal structure (red). **(B)** Compound_70 wrongly fitted in the pocket by CaverDock (green) with the original size of the tunnel. The overall position is similar, but orientation of the compound is incorrect. **(C)** Compound_70 correctly fitted in the pocket with the increased size of the tunnel. CaverDock (green) predicted a pose in equivalent position to the original crystal structure (red).

We calculated the RMSD between the positions of bound inhibitors and the lower-bound CaverDock snapshots. We report the lowest RMSDs and the RMSDs for the lowest energy conformations in Supplementary Table S1. Validation of CaverDock in terms of reproducibility of experimental structures of enzyme-inhibitor complexes revealed that the tool identified proper location and configuration in a vast majority (29 out of 34) cases. We show the example of correct fit in Figure 4A. In the case of compound_11, compound_19, compound_38, and compound_70 the correct pose was found by CaverDock but was not correctly identified. A different pose with the lowest energy was picked. In the case of the compound_18, CaverDock failed to find the correct conformation both for the closest and the lowest energy case. The high RMSDs may be caused by incorrect orientation of the ligand and also by the location of the inhibitor. The conformations of inhibitors which are deeper in the protein structure and are out of the tunnel may become unreachable for CaverDock since the ligand is always spatially constrained to the disks.

We experimented with the settings of CaverDock and recalculated the trajectories for the five problematic cases. We found out that by extending the radius of discretized tunnel discs by 10 Å, CaverDock is able to explore deeper parts of the cavity since the ligand has more freedom for movement. The resulting changes of RMSD are shown in Supplementary Table S2. The RMSDs were lowered and the binding poses were improved tremendously in case of compound_18, compound_19, and compound_70. This improvement in geometry for the compound_70 is shown in Figures 4B,C. The lowest energy pose for the compound_38 was still not identified correctly. Based on these findings, we decided to implement the tunnel extension for our future CaverDock calculations since the improvements were substantially beneficial.

#### Energy Analysis of N-HSP90 HOLO and APO Forms

CaverDock was used to analyze the unbinding of inhibitors from corresponding HOLO structures. We studied 32 cases with available kinetic data (Kokh et al., 2018). Selected energy values were extracted from the energy profiles: the energy minimum close to the start of the trajectory corresponding with the ligand-bound in the active site (E_Bound_) and the energy at the tunnel mouth—the last disk of the original tunnel—related with the surface-bound ligand (E_Surface_). In this specific case, there were no visible barriers as shown in the energy profiles Supplementary Figure S1. Therefore, we had to use the difference between bound and surface state, the ΔE_BS_ as possible energy barrier which needs to be overcome during the process of unbinding. We calculated the correlation between ΔE_BS_ and the experimentally measured values for k_on_, K_D_, and k_off_. We found a significant correlation of 0.53 for ΔE_BS_ with log(k_off_). Comparison of our results with the previously published correlation 0.63 for the computed relative residence times from molecular dynamics simulations, tcomp, with the measured residence times t_expt_ (t_expt_ = 1/k_off_), we confirmed that CaverDock is able to predict koff rates when HOLO structures are used with only a fraction of the computational effort.

We checked the previous findings from the HOLO dataset by simulating the complete set of inhibitors with the APO structure. We did not find any correlation in this case. This, together with no visible barriers in the CaverDock profiles and slow kinetic rates suggests conformational changes in the protein during the binding and unbinding of the inhibitor molecules. Essential conformational change is missing in the APO structure forcing the molecules to bind differently when simulated by CaverDock. Development of the new version of CaverDock that will be taking into account protein backbone dynamics is currently on-going in our laboratory.

### Screening of Inhibitors

**Figure 5:**
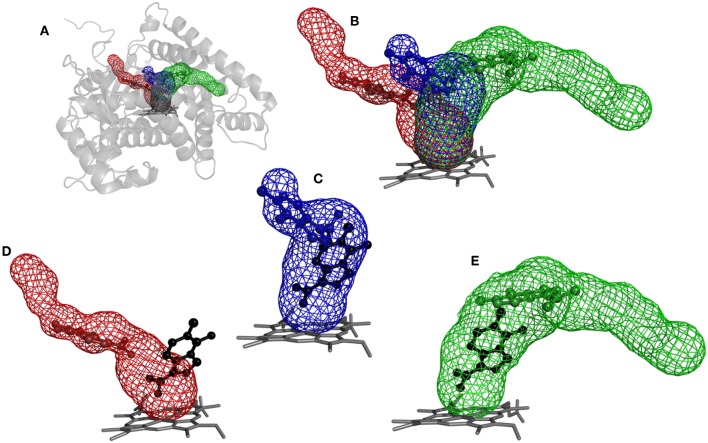
Structure of cytochrome P450 17A1 with tunnels calculated using Caver and binding poses obtained by CaverDock. **(A)** Global view of tunnels—tunnel 1 (blue), tunnel 2A (red), tunnel 3 (green). **(B)** View of tunnels with no protein visualized. Each tunnel has the ligand bound in the minimum obtained from the CaverDock continuous trajectory calculation. **(C)** Tunnel 1 and minimum binding energy pose obtained from CaverDock (blue) and AutoDock Vina (black). **(D)** Tunnel 2A and minimum binding energy pose obtained by CaverDock (red) and AutoDock Vina (black). **(E)** Tunnel 3 and minimum binding energy pose obtained by CaverDock (green) and AutoDock Vina (black).

**Figure 6:**
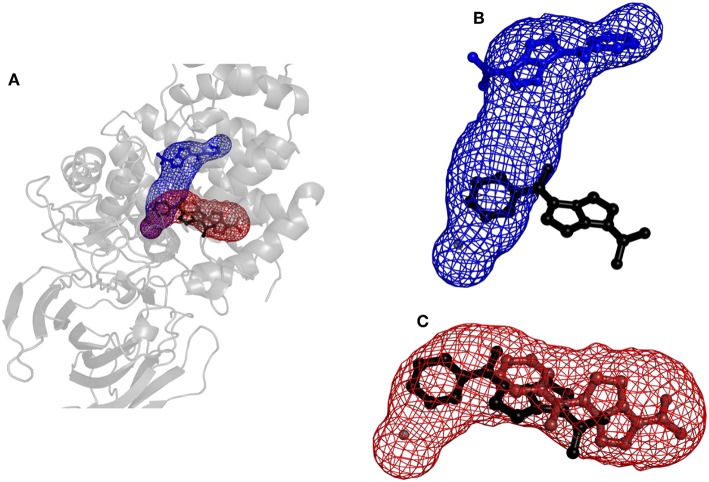
Structure of protein leukotriene A4 hydrolase/aminopeptidase with tunnels calculated using Caver and binding poses obtained by CaverDock. **(A)** Global view with tunnels LTA4 (blue) and PGP (red). **(B)** Tunnel LTA4 and minimum binding energy pose represented by balls and sticks obtained from CaverDock (blue) and AutoDock Vina (black). **(C)** Tunnel PGP and minimum binding energy pose represented by balls and sticks obtained from CaverDock (red) and AutoDock Vina (black).

The purpose of this analysis was to test whether CaverDock (Filipovic et al., 2019; Vavra et al., 2019) could be used for virtual screening. After deciding on the targets and libraries of compounds to use, we analyzed the tunnels for both targets. First, we choose the tunnels according to their ranking given by Caver and information from the literature and then used CaverDock to move the ligands from the outside of the proteins to the active site. Next, we performed virtual screening with the same libraries and targets using AutoDock Vina. It is worth noting that there was a large difference in the exhaustiveness used between the two programs: an exhaustiveness of thirty was used with Autodock Vina, whereas an exhaustiveness of one was used with CaverDock to keep the run time as short as possible. We showed that CaverDock provided new insights into the receptor ligand affinity. We also showed that CaverDock was a computationally cheap method with low run times. We studied 5 tunnels in the two proteins: three tunnels in cytochrome P450 17A1 (Figure 5) and two tunnels in leukotriene A4 hydrolase/aminopeptidase (Figure 6). Tunnel 1 was much shorter than the other two tunnels studied in cytochrome P450 17A1 (Table 2). It also had a narrow mouth when compared to the rest of the tunnel, but it was still wider than tunnel 3. Tunnel 2A was the most sinuous tunnel of the three, with more twists than the other two tunnels. However, they were not as sharp as the turn in tunnel 3. Tunnel 3 had a sharp turn halfway through the tunnel. It was narrow at the entrance of the protein, but after the turn widened sufficiently to allow a bulky inhibitor like Abiraterone to bind to the heme-group, as in the crystal structure.

**Table 2:** Summary of data for tunnels in the target proteins.

	**Cytochrome P450 17A1**			**Leukotriene A4 hydrolase/aminopeptidase**	
	**Tunnel 1**	**Tunnel 2A**	**Tunnel 3**	**Tunnel LTA4**	**Tunnel PGP**
Size of library	105	105	105	54	54
Continuous	41 (39.0%)	42 (40.0%)	42 (40.0%)	20 (37.0%)	21 (38.9%)
Lower bound	100 (95.2%)	91 (86.7%)	93 (88.6%)	48 (88.8%)	50 (92.6%)
Only lower bound	49 (46.7%)	59 (56.2%)	51 (48.6%)	28 (51.8%)	29 (53.7%)
Stopped at bottleneck	5 (4.8%)	14 (13.3%)	11 (10.5%)	6 (11.1%)	4 (7.4%)
Time average	41 min 50 s	68 min 59 s	52 min 11 s	36 min 17 s	22 min 23 s
Highest time	272 min 42 s	136 min 4 s	269 min 38 s	73 min 13 s	47 min 6 s
Lowest time	4 min 1 s	14 min 10 s	5 min 22 s	2 min 59 s	1 min 20 s
Length (Å)	15.1	24.9	28.2	20.4	25.4
Curvature (Å)	1.2	1.4	1.4	1.3	1.2
Maximum bottleneck (Å)	1.4	1.3	1.3	1.9	1.7
Average ligand RMSD lower bound docking (Å)	3.0	5.3	1.9	5.4	2.8
Average ligand RMSD continuous docking (Å)	6.8	10.8	10.5	11.2	6.8

The tunnels are modeled with the drug Temozolomide in both the CaverDock (Supplementary Video) and AutoDock Vina virtual screening (Figure 5). CaverDock yielded a minimum binding energy for a conformation inside the tunnel, rather than close to the heme group indicated by the AutoDock Vina calculation. The distance to the heme group was 10.3 Å with CaverDock for tunnel 3 and 2.6 Å with AutoDock Vina. Similar trends were observed for the other two tunnels. For tunnels 1 and 2A, CaverDock gave a minimum binding energy at 8.1 and 7.5 Å from the heme group, respectively. It should be noted that CaverDock was still able to bind the ligand to the heme group but at a higher energy than the conformations presented here. This result clearly demonstrates the value of the analysis of ligand binding and unbinding using CaverDock. Whereas, AutoDock Vina performs docking in a matrix box set by the user, CaverDock considers a continuous motion from the entrance of the tunnel to the active site, restrained to the tunnel found by Caver. It is also apparent in Figure 5, that tunnel 2A was deprecated by AutoDock Vina. Whereas, the closest nitrogen was bound to the heme group, the rest of the molecule was in a common area overlapping both tunnel 1 and tunnel 3. At the same time, the ligand was positioned away from tunnel 2A, with only a few atoms in the common space where all three tunnels overlapped. Despite these differences in the docking calculations, the minima binding energy obtained from CaverDock and binding affinity obtained from AutoDock Vina showed no significant differences for the case presented here. A complete data table comparing the AutoDock Vina and CaverDock virtual screening is presented in the Supporting Information.

Results for leukotriene A4 hydrolase/aminopeptidase are shown in Figure 6. Comparing tunnel LTA4 (blue) with tunnel PGP (red), only slight differences were discerned in the sizes of the tunnels. Tunnel PGP had a sharp turn, whereas tunnel LTA4 did not. Tunnel LTA4 had a higher overall curvature than tunnel PGP. Both tunnels are presented with the minimum binding energy pose obtained from CaverDock with the drug Ketorolac. Ketorolac was not bound to the zinc atom in the active site for both studied cases. AutoDock Vina yielded a conformation with the drug molecule at a distance of 4.8 Å from the zinc atom and clearly docked in the tunnel PGP, with only one ring in the common area overlapping both tunnels (Figure 6). Using CaverDock, the minima were even farther away from the zinc in the active site: the distance in tunnel PGP was 8.8 Å and in tunnel LTA4 11.8 Å. When the drug molecule is bound in tunnel LTA4, higher energetic barriers were obtained. It is known that pro-inflammatory mediator biosynthesis occurs through tunnel LTA4 and that the inhibitor Pro-Gly-Pro enters and exerts its effects through a different tunnel (Sanson et al., 2011; Čolović et al., 2013).

One of the main objectives of this study was to assess the computational costs of a project with the novel computational tool. In total, 105 drugs were docked to the three tunnels in cytochrome P450 17A1 (Table 2). From these, CaverDock was able to finish a continuous (upper bound) trajectory calculation for 39.7% of the drugs and a discontinuous (lower bound) trajectory for 90.2%. On average, the drugs were not able to overcome the bottlenecks 9.8% of the time. This result does not mean that the calculation failed but that the ligand was not able to pass through the rigid receptor. Values were also similar for leukotriene A4 hydrolase/aminopeptidase. The length of the five tunnels studied ranged from 15.1 to 28.2 Å, the curvature of the tunnels ranged from 1.2 to 1.4 Å and bottlenecks ranged from 1.3 to 1.9 Å. These differences in length, curvature and bottlenecks yielded very different tunnels and tunnel shapes, as evident in Figures 5, 6. The approach presented here constitutes a computationally low-cost method for virtual screening with a run time average of 2,660 s (~44 min). Moreover, when the upper bound calculation was turned off, the lower bound results could be completed within several minutes using a computer with 4 processors. Note that each calculation runs independently, allowing users with sufficient computing power to perform a virtual screening protocol on a full library in a parallel manner.

**Figure 7:**
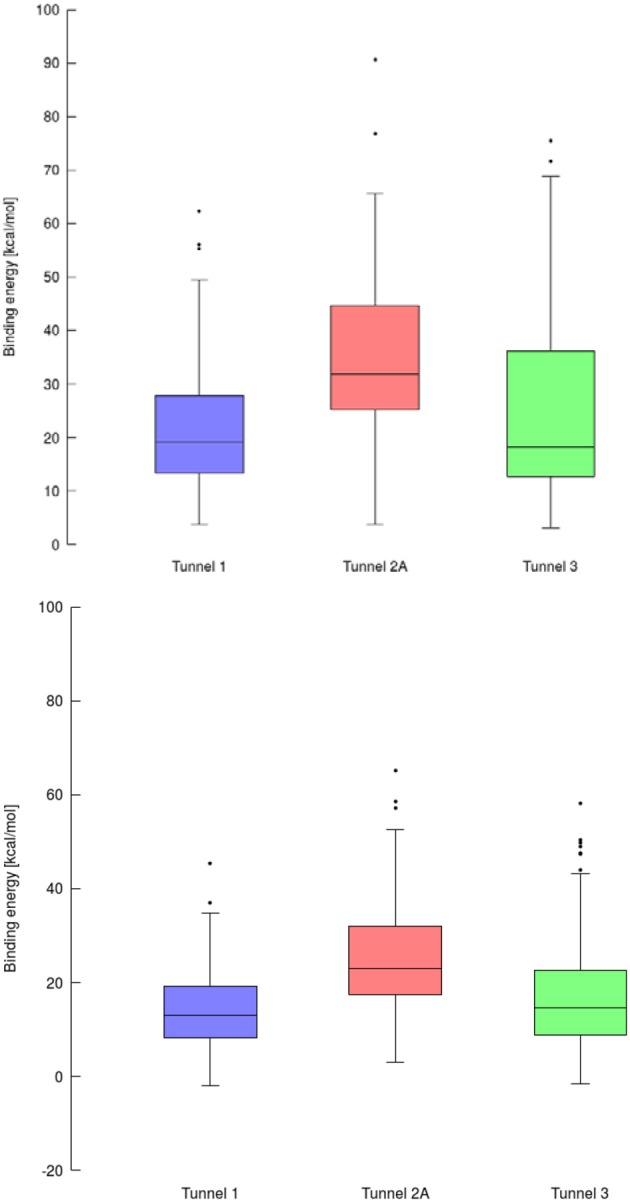
Box and Whiskers plot representing the maxima (energy barriers) for each continuous (up) and lower bound (down) trajectories obtained for cytochrome P450 17A1. Outlying values are indicated by circles.

**Figure 8:**
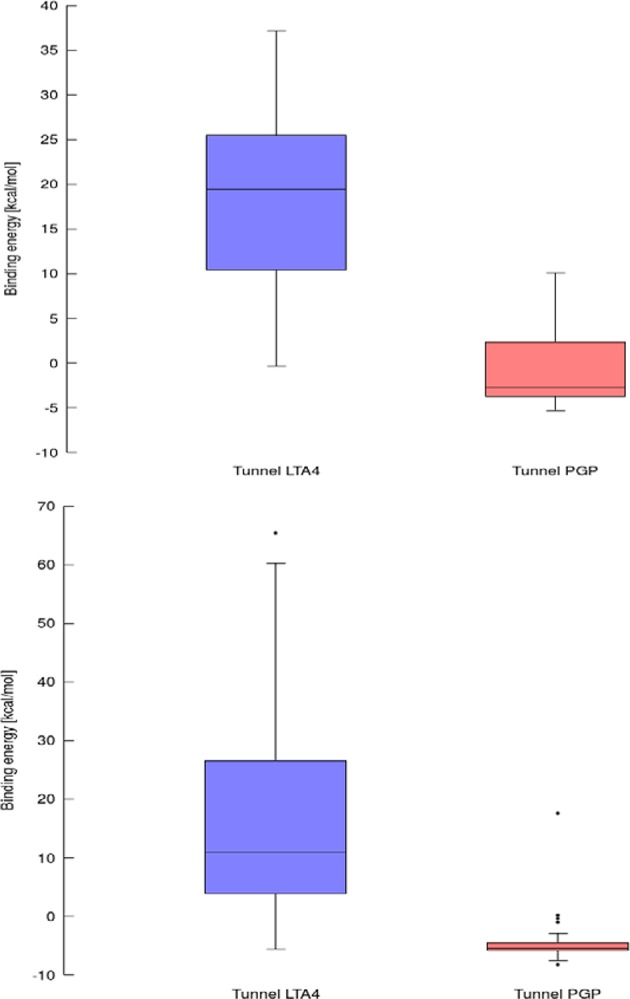
Box and Whiskers plot representing the maxima (energy barriers) for continuous (up) and lower bound (down) trajectories obtained for leukotriene A4 hydrolase/aminopeptidase. Outlying values are indicated by circles.

Using data obtained from a virtual screening campaign, it is possible to analyze a functionally important tunnel for a given target and set of drugs. Although it is not always easy to select a preferred tunnel, it may be possible to identify tunnels that are not favored. We found that in the case of cytochrome P450 17A1, the jobs finished successfully with a continuous trajectory and tunnel 2A had higher barriers than the other two tunnels (Figure 7), therefore it is not preferred. However, we could not determine which of the two remaining tunnels 1 and 3 would be better to consider in a drug design project since there was no statistically significant difference in the energy barriers. Possibly both tunnels can be explored by ligands during their (un)binding. The results were more conclusive for leukotriene A4 hydrolase/aminopeptidase, as shown by the differences in energy barriers (Figures 7, 8). In the case of the continuous (upper-bound) calculation, the drug molecule was taken through one smooth trajectory with the possibility of backtracking if it encountered a bottleneck. Backtracking allowed the drug to find a more favorable conformation to overcome the bottleneck. In the case of the lower-bound calculation, once the drug encountered a bottleneck, it was allowed to flip in order to find a more suitable conformation on the other side of the bottleneck, while the point being dragged through the discs of the tunnel was kept constant (Figure 8). This trajectory always yielded a lower energy value for the barrier because, by definition, the bottleneck was easier to overcome. On the other hand, lower time demands and similar results make the lower bound calculation very powerful for virtual screening.

## Conclusions

Our results demonstrate that CaverDock is applicable for screening of large libraries of potential inhibitors. It provides information on binding and unbinding processes. The tool estimates a profile of potential energies and calculates respective trajectories without the need for time-demanding molecular dynamics simulations. Setting up a calculation using CaverDock is simple and comprises five steps: (i) definition of a receptor, (ii) definition of the ligands, (iii) calculation of tunnels using Caver, (iv) screening of un/binding trajectories, and (v) data analysis. The tool is accompanied by a user manual that explains the setting up of calculations as well as troubleshooting. A standalone version of CaverDock with detailed documentation is available at https://loschmidt.chemi.muni.cz/caverdock/. The automated version of CaverDock is available via the web https://loschmidt.chemi.muni.cz/caverweb/.

The dynamics of side chains lining the protein tunnels and channels can be described to a certain level with the current implementation of CaverDock. Making residue side chains flexible increases calculation times but ultimately considers protein dynamics. We concluded that simulations employing a large number (>4) of flexible amino acid residues may cause undesirable steric clashes. Thus, we advise that results obtained with flexible residues should be interpreted carefully using biochemical intuition when analyzing calculated trajectories and energy profiles. Implementation of a more thorough protocol to address protein flexibility is on-going in our laboratory. CaverDock calculations can be extended to ensembles of protein structures. Particularly challenging is the trade-off between rigorous description of flexible systems and time demands connected with such calculations. Structural comparison of complexes obtained by CaverDock with those determined by crystallographic analysis revealed that we were able to predict the correct poses for a vast majority of inhibitors. The comparison of our profile of potential energies with the rates obtained by kinetic results yields a correlation of 0.53 whereas the more computational expensive molecular dynamics simulation had a correlation of 0.63. Prediction accuracy can be potentially improved by proper treatment of backbone flexibility.

Our study demonstrates that CaverDock is sufficiently fast to screen even large libraries of ligands. Calculation of rigid trajectories using 4 processors took on average 53 min per molecule with 90% successfully calculated cases. Bulky or very flexible ligands take more time, but some of these large ligands may not be able to access the active site via the studied access tunnels. Although it takes longer to perform a CaverDock calculation than a pure virtual screening of ligand binding to the active site with AutoDock Vina, CaverDock provides more data, which may be useful in rational drug design projects. Information on the bottlenecks and energy required for ligands to pass through these narrowed parts of the access tunnel could be useful for medicinal chemists. CaverDock was able to correctly identify tunnels in the proteins explored by the inhibitors included in our screening campaigns.

In summary, we have shown that CaverDock is a robust and ready-to-use software that can be employed in screening campaigns of important pharmacological targets. CaverDock analysis may be a useful complement to virtual screening campaigns carried out using traditional docking tools.

## Data Availability Statement

The datasets generated for this study can be found in the https://loschmidt.chemi.muni.cz/data/caverdock/pinto_2019_suppl/.

## Author Contributions

GP and OV carried out the computational work and wrote the manuscript. JF developed the software. DB and JD designed the study. All authors contributed to interpretation of the data, revision of the manuscript, and have given approval of its final version.

### Conflict of Interest

The authors declare that the research was conducted in the absence of any commercial or financial relationships that could be construed as a potential conflict of interest.

## Supplementary Material

Supplementary Video Lower bound calculation of Temozolomide through Tunnel 3 of cytochrome P450 17A1.
