# Supplementary material for: Fast Screening of Inhibitor Binding/Unbinding Using Novel Software Tool CaverDock
Source: Front Chem. 2019 Oct 29;7:709. doi: 10.3389/fchem.2019.00709 (PMC6828983; doi:10.3389/fchem.2019.00709)
Supplement: Supplementary file 1 [file Data_Sheet_1.pdf]

Supporting Information

Fast Screening of Inhibitor Binding/Unbinding using Novel Software Tool CaverDock

Gaspar P. Pinto[1,2,#], Ondrej Vavra[1,2,#], Jiri Filipovic[3], Jan Stourac[1,2], David Bednar[1,2,*], Jiri Damborsky[1,2,*]

[1] Loschmidt Laboratories, Department of Experimental Biology and RECETOX, Faculty of Science, Masaryk University, Brno, Czech Republic

[2] International Centre for Clinical Research, St. Anne's University Hospital Brno, Brno, Czech Republic

[3] Institute of Computer Science, Faculty of Science, Masaryk University, Brno, Czech Republic

[#] These authors contributed equally to this work.

* Correspondence: David Bednar, 222755@mail.muni.cz; Jiri Damborsky, jiri@chemi.muni.cz

Here we present the details for the protein dataset used for the simulation with flexible side chains. Furthermore, we present the tables and plots for the dataset used to compare the results from CaverDock with laboratory experiments. We then present the plots with the results obtained from the CaverDock continuous trajectory calculations for the three tunnels of the cytochrome P450 17A1 and two tunnels of the leukotriene A4 hydrolase/aminopeptidase. We also show radius changes along the tunnel obtained by Caver. Finally, we present the root mean square deviations for every finished CaverDock calculation (lower bound and continuous trajectory) and compare them with the results obtained with the AutoDock Vina.

Attached electronic supplementary material files works with most of the browsers except Chrome, which does not allow running external scripts, that is essential for 3D visualization using JSmol. Electronic supplementary material is available also from this web site and works for all browsers:

https://loschmidt.chemi.muni.cz/data/caverdock/pinto_2019_suppl/

Details on the proteins used for the simulations with flexible side chains:

Acetylcholinesterase AChE plays an important role in cholinergic synapses for both the peripheral and central nervous systems. These synapses terminate the transmission of impulses by hydrolyzing the natural substrate acetylcholine. The first generation of Alzheimer drugs specifically targeted this enzyme. As cholinergic neurons are widespread across animals, anti-AChE have also been used as pesticides, agricultural insecticides and even nerve gases in warfare (Čolović et al., 2013; Silman and Sussman, 2005). We used enzyme-inhibitor complex PDB-ID 2XI4 (Sanson et al., 2011) for this work. As for the other enzymes, we deleted the inhibitor from the structure before starting the CaverDock protocol.

Cholesteryl ester transfer protein CETP controls the transport of cholesteryl ester from high-density lipoproteins (HDLs) to low and very low-density lipoproteins (LDLs and VLDLs) (Geller and Billiar, 1998). High concentrations of LDLs and VLDLs and low concentrations of HDLs are considered to be good predictors of cardiovascular risks. Inhibition of CETP is being considered as a novel approach to prevent these risks (Cannon, 2011; Dullaart et al., 2007; Joy and Hegele, 2008). The structure PDB-ID 4EWS (Liu et al., 2012) was used, which is a complex with the drug Torcetrapib. CETP was the only protein with a channel considered: the substrate enters on one side of the protein, travels into the active site and the products exit through another side of the protein.

Nitric oxide synthase iNOS synthesizes nitric oxide from L-arginine. Its excess has been implicated in inflammatory bowel disease, rheumatoid arthritis, stroke thrombosis and general inflammation (Bian and Murad, 2003; Duncan and Heales, 2005; Nathan, 2004). The structure of iNOS was that of PDB-ID 3EBF (Garcin et al., 2008) complexed with inhibitor (3R)-3-[(1,2,3,4-tetrahydroisoquinolin-7-yloxy)methyl]-2,3-dihydrothieno[2,3-f][1,4]oxazepin-5-amine, with code name 332.

Metal-dependent deacetylase LpxC is a zinc-dependent metalloprotein that promotes the deacetylation of UDP-3-O-N-acetylglucosamine, which is an essential step in the biosynthesis of lipid A. Inhibitors of this enzyme have been studied as potentially new antibiotic targets to combat the increase in multi-drug resistant bacteria. Although some narrow spectrum inhibitors have been approved by the FDA, broad spectrum antibiotic inhibitors have only been approved already during in this decadee last ten years (Arolas et al., 2011). LpxC inhibitors represent a class of antibiotics that act not by killing bacteria but by downregulating hindering instead their ability of bacteria to start phagocytosis and thus to induce sepsis (Chemonges, 2014; Tomaras et al., 2014; Whittington et al., 2003). We used the structure PDB-ID 3NZK (Cole et al., 2011) complexed with the inhibitorwith inhibitor N-{(1S,2R)-2-hydroxy-1-[(hydroxyamino)carbonyl]propyl}-4-{[4-(morpholin-4-ylmethyl)phenyl]ethynyl}benzamide, with code name C90.

Matrix metalloproteinase-13 MMP-13 is a metalloprotein with similar motif to leukotriene HEXXH. However, MMP-13 has a longer motif present in the superfamily of endopeptidases, HEXXHXXGXXH (Stöcker and Bode, 1995). MMP13 belongs to the collagenase subfamily. It is the most active protein of the subfamily in cleaving collagen, making it a preferred target for the design of inhibitors to combat osteoarthritis (Wasserman, 2005). We used the structure PDB-ID 1XUD (Engel et al., 2005), which was complexed with inhibitor N,N'-bis(4-fluoro3-methylbenzylL)pyrimidine-4,6-dicarboxamide, with code name PB4.

Supplementary Table S1. Root mean square deviation (RMSD) values for the structural analysis of HOLO dataset.

Complex	PDB ID	RMSD (Å) of closest conformation to bound inhibitors	RMSD (Å) of lowest energy conformations
compound_01	2VCI	0.86	1.25
compound_02	2UWD	0.67	0.67
compound_03	2BSM	0.60	0.60
compound_04	5NYI	0.60	2.01
compound_05	5J2X	0.83	1.98
compound_06	6F1N	0.81	1.87
compound_07	6ELO	0.31	0.31
compound_08	5J64	0.54	1.56
compound_10	6ELN	0.49	0.49
compound_11	5J20	0.50	8.30
compound_12	5J86	0.46	0.49
compound_13	5J9X	0.66	0.67
compound_14	6ELP	0.26	0.29
compound_15	5J27	0.41	0.45
compound_17	5LRZ	3.14	3.58
compound_18	5LR7	9.83	9.88
compound_19	2YKI	2.65	9.57
compound_20	5LQ9	0.80	0.84
compound_21	5LS1	0.84	1.12
compound_22	5T21	0.94	1.31
compound_35	6EYA	1.08	1.08
compound_36	5LO6	0.61	0.61
compound_37	5LNZ	1.10	1.31
compound_38	6EY8	1.15	5.49
compound_40	6EY9	0.39	1.29
compound_43	5OCI	1.26	1.51
compound_50	5ODX	1.07	1.10
compound_51	5NYH	0.44	0.44
compound_60	5OD7	0.48	0.51
compound_62	6EI5	0.44	0.77
compound_67	5LR1	0.42	0.42
compound_68	6EL5	0.48	1.71
compound_69	5LO5	1.13	1.13
compound_70	2YKJ	1.61	9.32

Supplementary Table S2. Improvement in root mean square deviation (RMSD) by using extended tunnel radius.

**Analysed case**	**Default settings**		**Extended tunnel radius**	
	**Lowest RMSD (Å)**	**Low Energy RMSD (Å)**	**Lowest RMSD (Å)**	**Low Energy RMSD (Å)**
compound_11	0.50	8.30	0.57	7.99
compound_18	9.83	9.88	1.27	2.18
compound_19	2.65	9.57	1.81	2.26
compound_38	1.15	5.49	0.43	5.59
compound_70	1.61	9.32	0.31	0.31

Supplementary Table S3. CaverDock energies for the HOLO dataset.

Ligand	E_{bound}	$E_{Surface}$	ΔE_{BS}	k_{off} [s^{-1}]	K_D [M^{-1}]	k_{on} [$M^{-1}s^{-1}$]	log(k_{off})	log(K_D)	log(k_{on})	t_{comp} [ns]	1 / k_{off}	log(1/k_{off})
compound_02	-8.6	-3.10	-5.5	0.0021	3.5E-09	580000	-2.68	-8.4559	5.7634	2.6	476.1905	2.6778
compound_03	-8.9	-4.20	-4.7	0.01	0.000000011	970000	-2.00	-7.9586	5.9868	2.2	100.0000	2.0000
compound_05	-7.9	-3.50	-4.4	0.014	2.66E-08	520000	-1.85	-7.5751	5.7160	0.7	71.4286	1.8539
compound_06	-7.4	-3.20	-4.2	0.109	0.000000547	199000	-0.96	-6.2620	5.2989	0.8	9.1743	0.9626
compound_07	-9.1	-3.60	-5.5	0.0634	0.000000514	123000	-1.20	-6.2890	5.0899	0.8	15.7729	1.1979
compound_08	-9.0	-2.80	-6.2	0.21	0.00000018	1200000	-0.68	-6.7447	6.0792	0.3	4.7619	0.6778
compound_10	-8.4	-3.80	-4.6	0.254	0.0000009	280000	-0.60	-6.0458	5.4472	0.4	3.9370	0.5952
compound_11	-10.7	-5.50	-5.2	0.00033	7.2E-10	215000	-3.48	-9.1427	5.3324	1.4	3030.3030	3.4815
compound_12	-9.5	-3.80	-5.7	0.00179	3.81E-09	472000	-2.75	-8.4191	5.6739	3.0	558.6592	2.7471
compound_13	-9.8	-4.10	-5.7	0.0017	0.000000023	70000	-2.77	-7.6383	4.8451	1.8	588.2353	2.7696
compound_14	-10.0	-3.80	-6.2	0.174	0.000000236	742000	-0.76	-6.6271	5.8704	0.3	5.7471	0.7595
compound_15	-8.4	-3.40	-5.0	0.0064	0.000000087	77000	-2.19	-7.0605	4.8865	3.3	156.2500	2.1938
compound_17	-12.3	-4.20	-8.1	0.000278	0.000000172	3060	-3.56	-6.7645	3.4857	1.5	3597.1223	3.5560
compound_18	-11.2	-4.30	-6.9	0.000189	4.66E-08	4770	-3.72	-7.3316	3.6785	4.6	5291.0053	3.7235
compound_19	-10.2	-4.80	-5.4	0.000285	3.61E-08	7770	-3.55	-7.4425	3.8904	4.0	3508.7719	3.5452
compound_20	-14.1	-4.00	-10.1	0.000136	8.48E-09	16200	-3.87	-8.0716	4.2095	4.8	7352.9412	3.8665
compound_21	-11.8	-4.20	-7.6	0.000485	3.95E-09	133000	-3.31	-8.4034	5.1239	3.6	2061.8557	3.3143
compound_22	-11.7	-3.00	-8.7	0.000765	2.4E-10	3580000	-3.12	-9.6198	6.5539	2.6	1307.1895	3.1163
compound_35	-11.3	-4.30	-7.0	0.0054	0.000000033	160000	-2.27	-7.4815	5.2041	2.2	185.1852	2.2676
compound_36	-9.7	-4.50	-5.2	0.00139	5.39E-08	25300	-2.86	-7.2684	4.4031	2.5	719.4245	2.8570
compound_37	-9.0	-4.00	-5.0	0.00201	5.96E-09	343000	-2.70	-8.2248	5.5353	4.6	497.5124	2.6968
compound_38	-9.9	-3.90	-6.0	0.0288	0.00000155	18600	-1.54	-5.8097	4.2695	1.0	34.7222	1.5406
compound_40	-10.0	-4.00	-6.0	0.0174	0.00000124	14200	-1.76	-5.9066	4.1523	1.0	57.4713	1.7595
compound_43	-11.2	-4.00	-7.2	0.000679	8.97E-09	83800	-3.17	-8.0472	4.9232	6.2	1472.7541	3.1681
compound_50	-10.3	-4.70	-5.6	0.000294	0.000000195	1650	-3.53	-6.7100	3.2175	6.7	3401.3605	3.5317
compound_51	-10.3	-4.60	-5.7	0.00243	1.87E-08	130000	-2.61	-7.7282	5.1139	3.6	411.5226	2.6144
compound_60	-12.7	-6.00	-6.7	0.000238	3.68E-09	64300	-3.62	-8.4342	4.8082	0.4	4201.6807	3.6234
compound_62	-11.9	-4.40	-7.5	0.00453	3.83E-08	121000	-2.34	-7.4168	5.0828	0.2	220.7506	2.3439
compound_67	-9.0	-3.40	-5.6	0.0256	2.47E-08	1260000	-1.59	-7.6073	6.1004	0.1	39.0625	1.5918
compound_68	-8.6	-3.40	-5.2	0.0331	0.000000133	248000	-1.48	-6.8761	5.3945	0.7	30.2115	1.4802
compound_69	-9.4	-5.10	-4.3	0.429	0.00000134	175000	-0.37	-5.8729	5.2430	1.0	2.3310	0.3675
compound_70	-11.2	-5.20	-6.0	0.000989	0.000000095	10400	-3.00	-7.0223	4.0170	0.5	1011.1223	3.0048

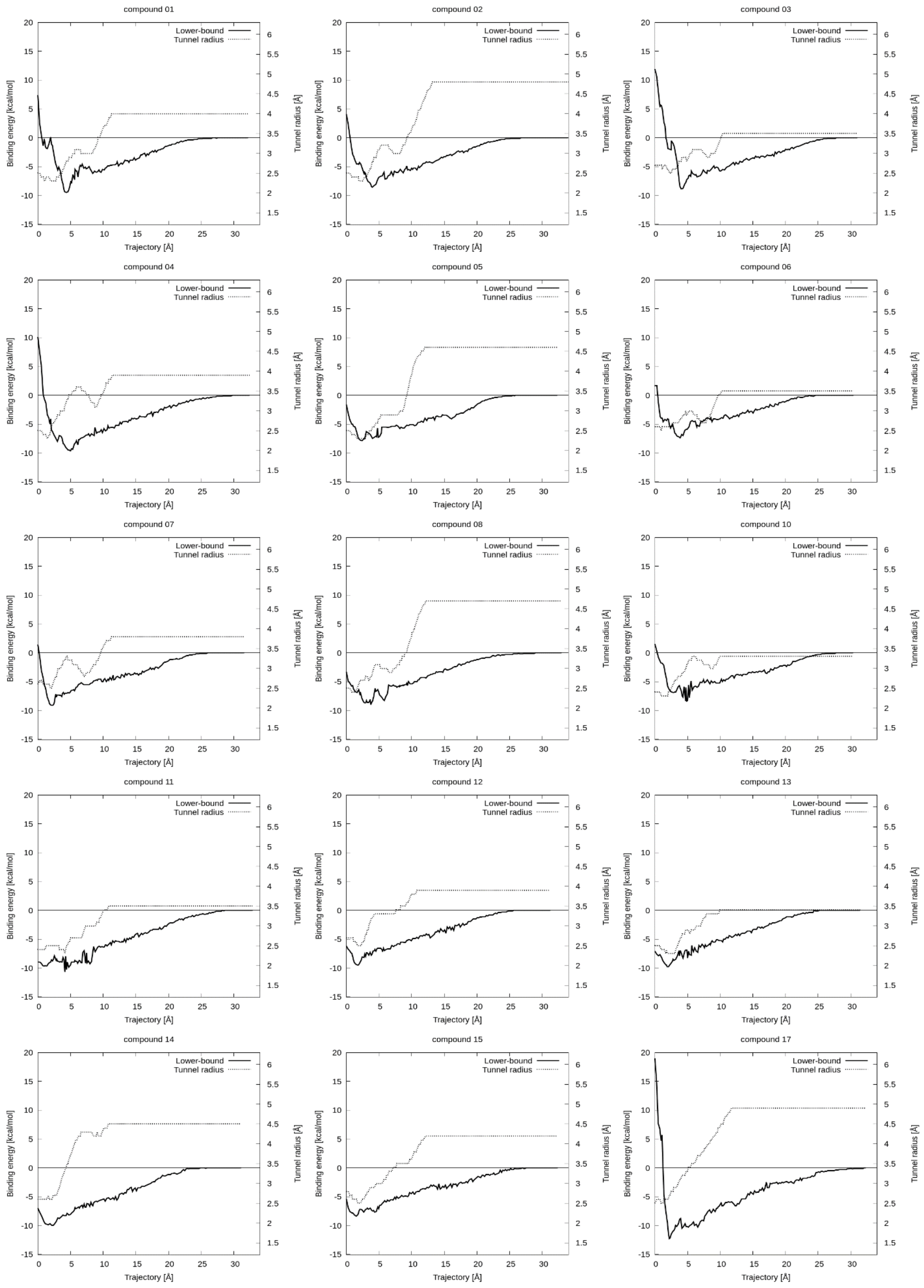
compound 01
compound 02
compound 03
compound 04
compound 05
compound 06
compound 07
compound 08
compound 10
compound 11
compound 12
compound 13
compound 14
compound 15
compound 17
Lower-bound
Tunnel radius
Binding energy [kcal/mol]
Tunnel radius [Å]
Trajectory [Å]

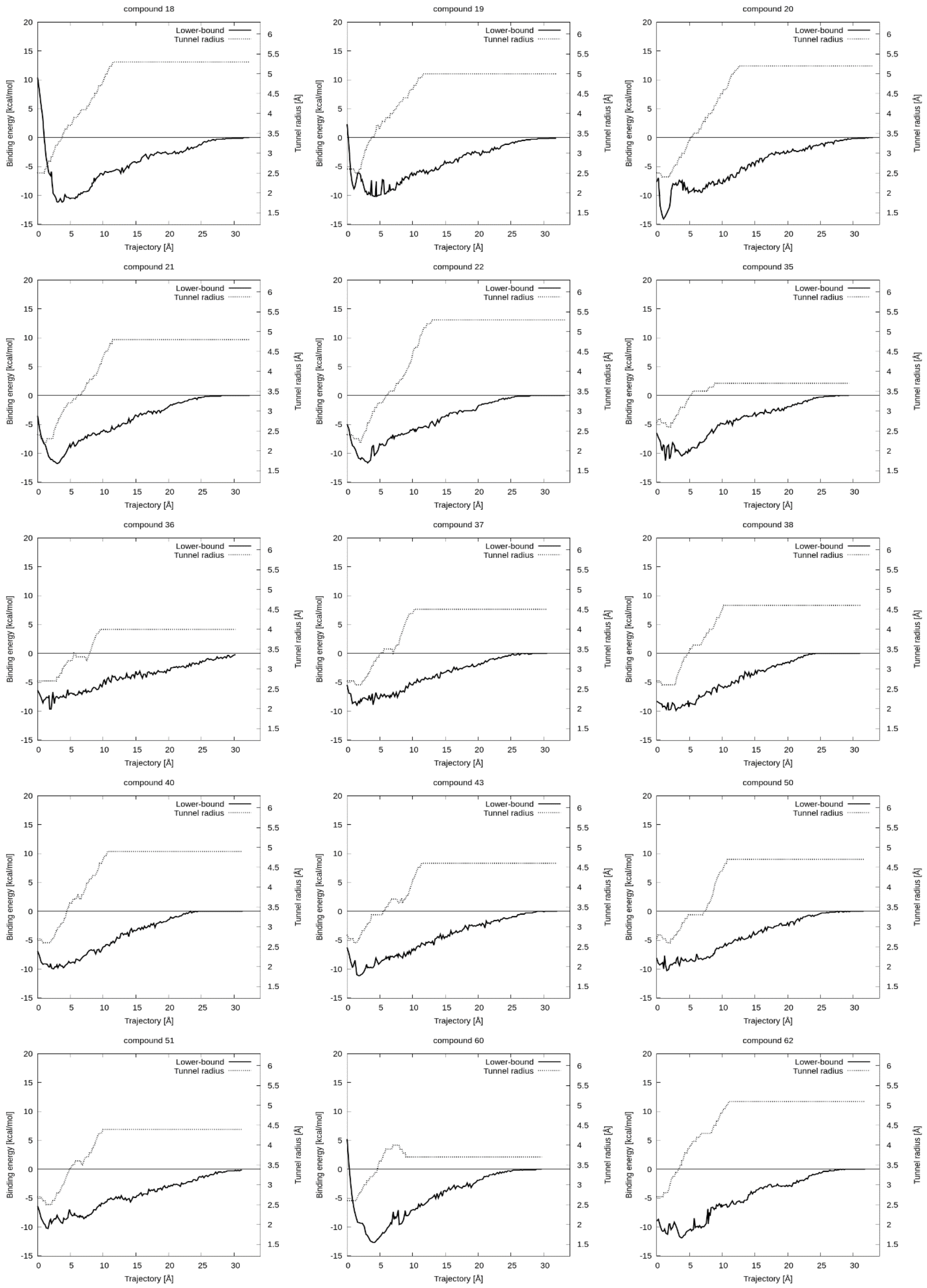
compound 18
compound 19
compound 20
compound 21
compound 22
compound 35
compound 36
compound 37
compound 38
compound 40
compound 43
compound 50
compound 51
compound 60
compound 62
Lower-bound
Tunnel radius
Binding energy [kcal/mol]
Tunnel radius [Å]
Trajectory [Å]

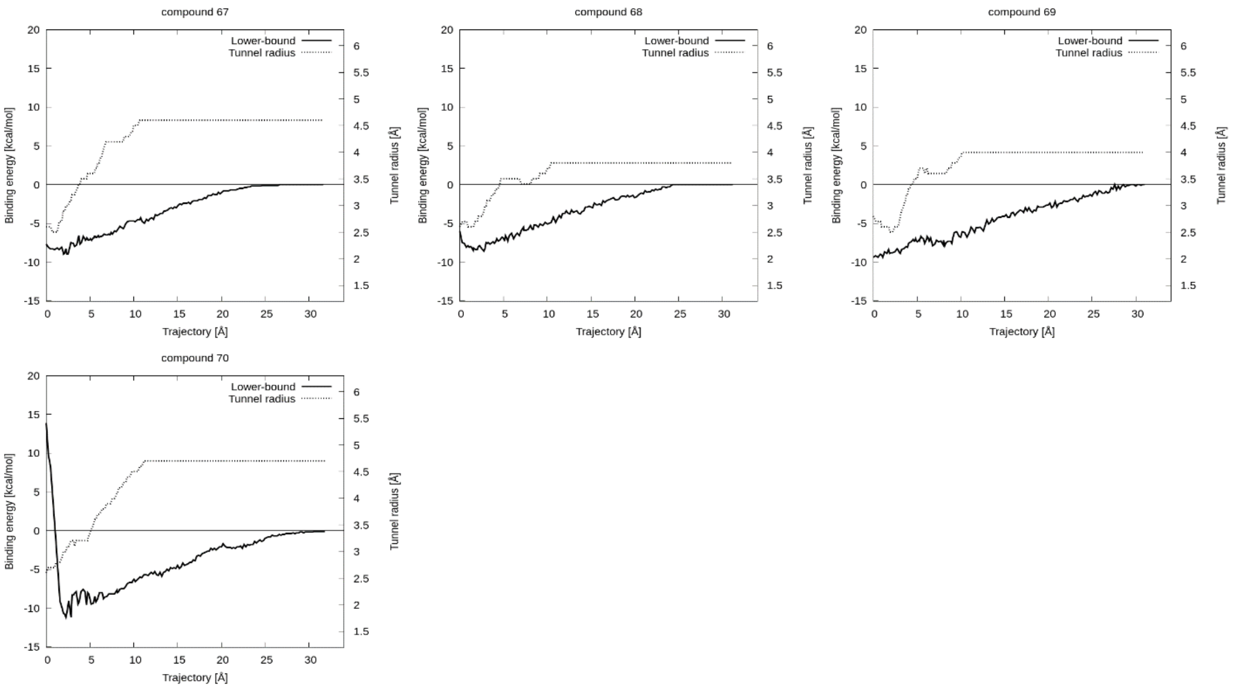

Supplementary Figure S1. Profile of potential energies of the dataset used to compare the results from CaverDock calculation and laboratory experiments.

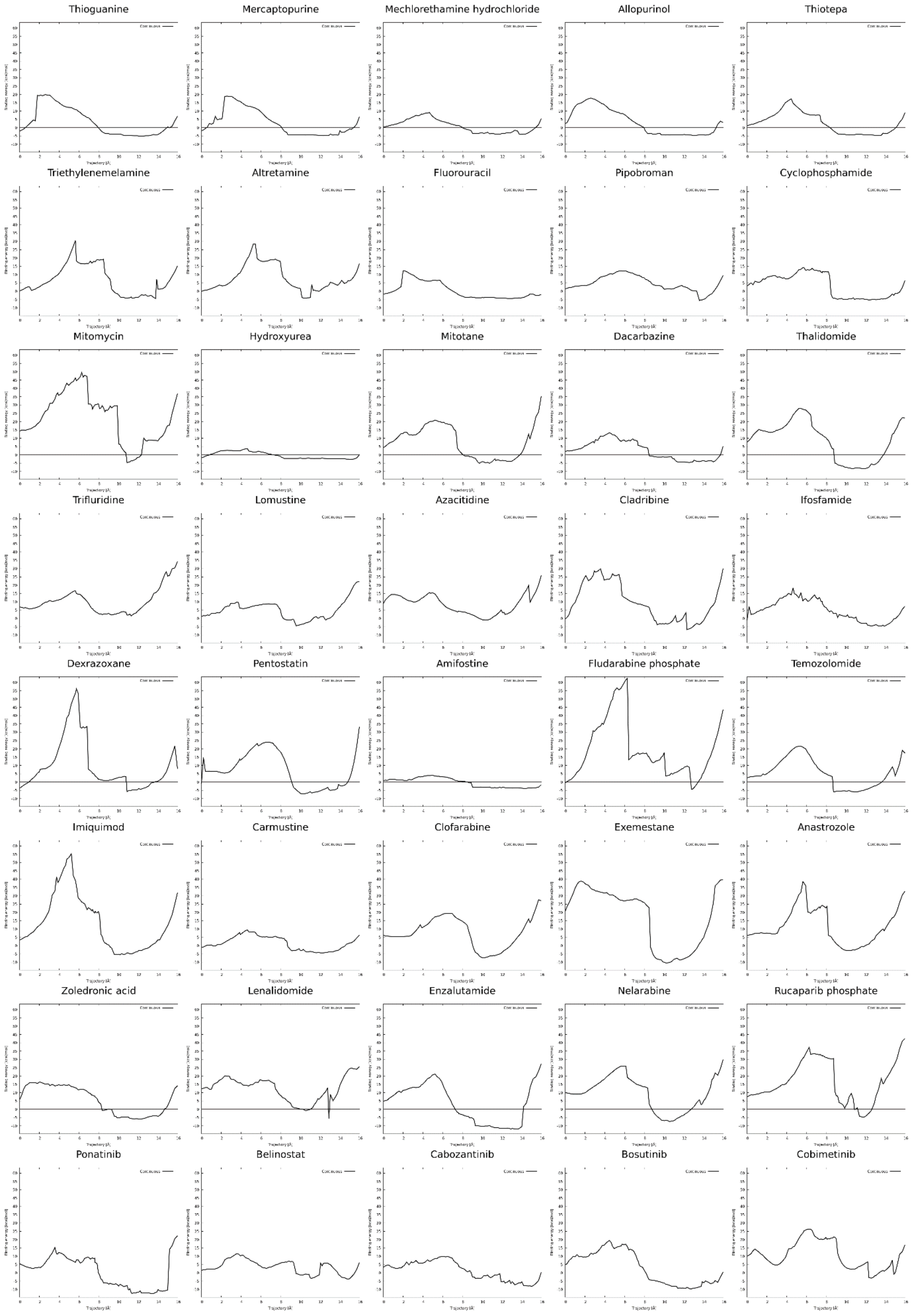
Thioguanine
Mercaptopurine
Mechlorethamine hydrochloride
Allopurinol
Thiotepa
Triethylenemelamine
Altretamine
Fluorouracil
Pipobroman
Cyclophosphamide
Mitomycin
Hydroxyurea
Mitotane
Dacarbazine
Thalidomide
Trifluridine
Lomustine
Azacitidine
Cladribine
Ifosfamide
Dexrazoxane
Pentostatin
Amifostine
Fludarabine phosphate
Temozolomide
Imiquimod
Carmustine
Clofarabine
Exemestane
Anastrozole
Zoledronic acid
Lenalidomide
Enzalutamide
Nelarabine
Rucaparib phosphate
Ponatinib
Belinostat
Cabozantinib
Bosutinib
Cobimetinib

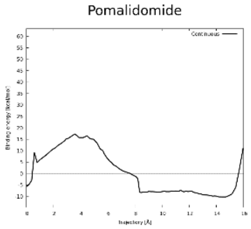

Supplementary Figure S2. Binding energy plots for all successfully finished jobs for the continuous trajectory calculation for the tunnel 1 of cytochrome P450 17A1. Binding energy (in kcal/mol) is plotted against the tunnel length (in Å).

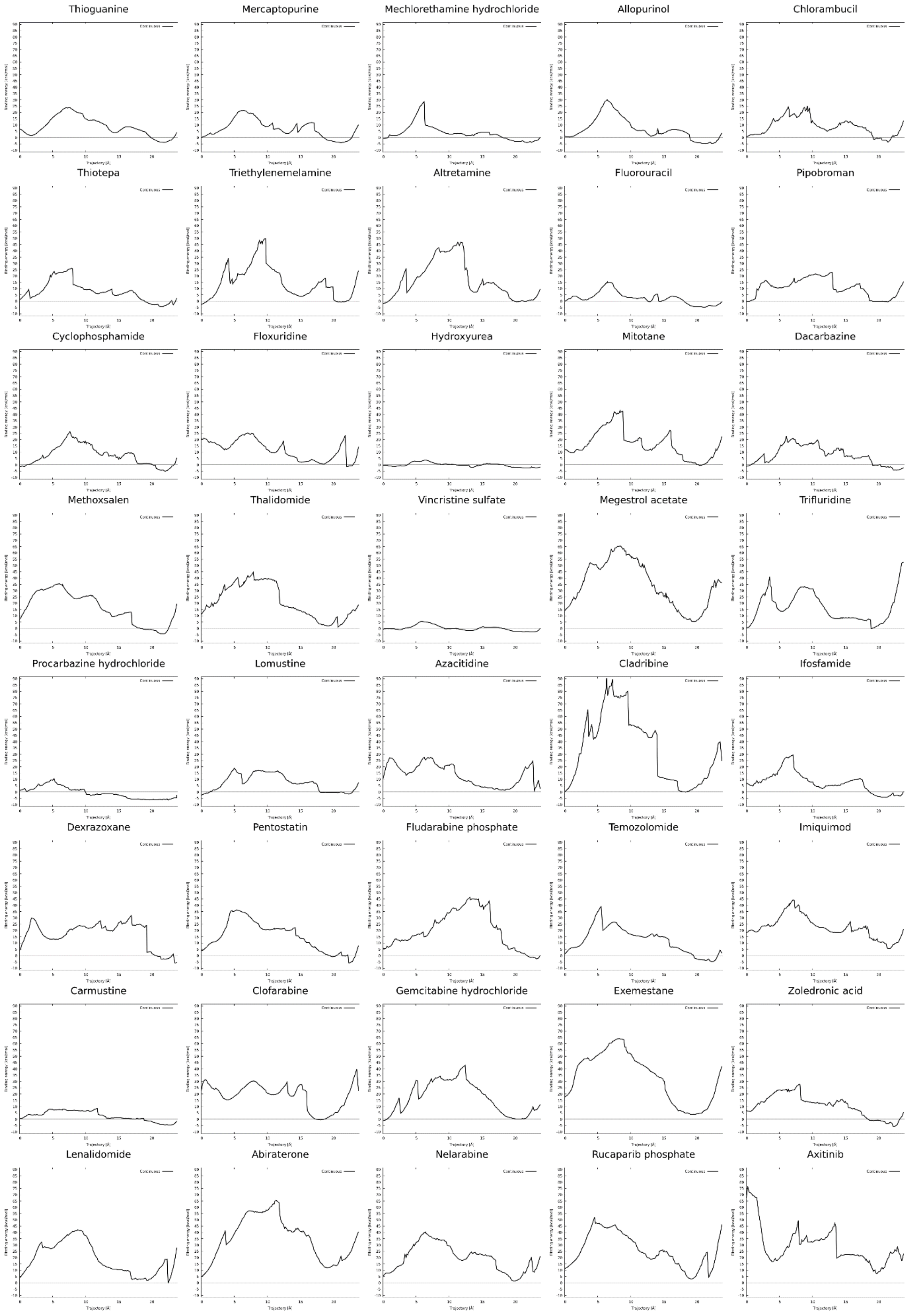
Thioguanine
Mercaptopurine
Mechlorethamine hydrochloride
Allopurinol
Chlorambucil
Thiotepa
Triethylenemelamine
Altretamine
Fluorouracil
Pipobroman
Cyclophosphamide
Floxuridine
Hydroxyurea
Mitotane
Dacarbazine
Methoxsalen
Thalidomide
Vincristine sulfate
Megestrol acetate
Trifluridine
Procarbazine hydrochloride
Lomustine
Azacitidine
Cladribine
Ifosfamide
Dexrazoxane
Pentostatin
Fludarabine phosphate
Temozolomide
Imiquimod
Carmustine
Clofarabine
Gemcitabine hydrochloride
Exemestane
Zoledronic acid
Lenalidomide
Abiraterone
Nelarabine
Rucaparib phosphate
Axitinib

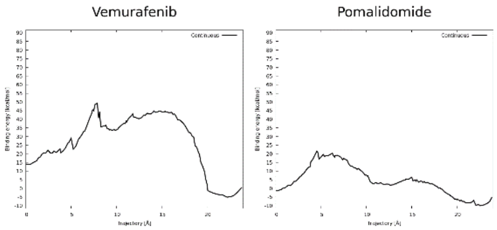

Supplementary Figure S3. Binding energy plots for all successfully finished jobs for the continuous trajectory calculation for the tunnel 2A of cytochrome P450 17A1. Binding energy (in kcal/mol) is plotted against the tunnel length (in Å).

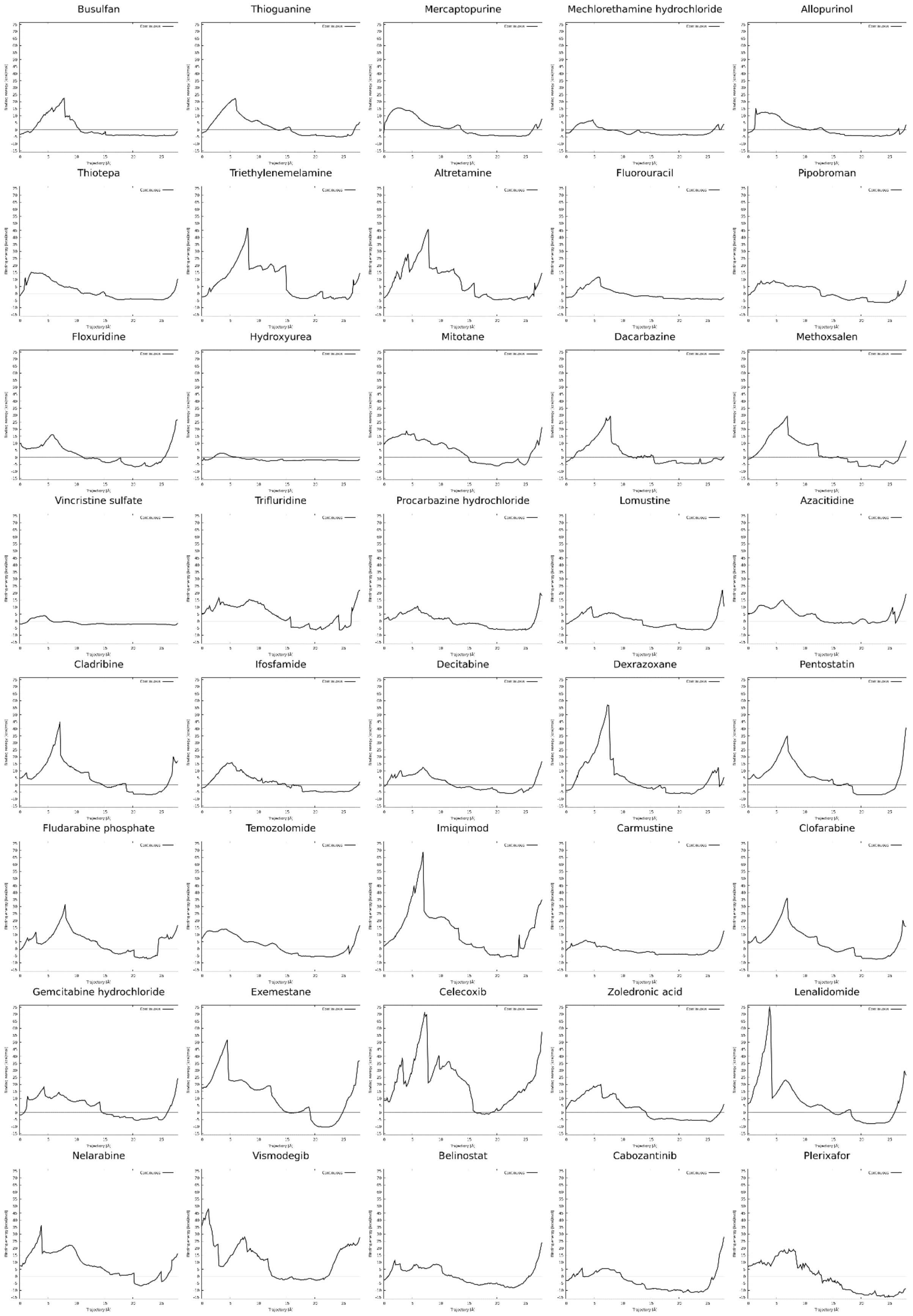
Busulfan
Thioguanine
Mercaptopurine
Mechlorethamine hydrochloride
Allopurinol
Thiotepa
Triethylenemelamine
Altretamine
Fluorouracil
Pipobroman
Floxuridine
Hydroxyurea
Mitotane
Dacarbazine
Methoxsalen
Vincristine sulfate
Trifluridine
Procarbazine hydrochloride
Lomustine
Azacitidine
Cladribine
Ifosfamide
Decitabine
Dexrazoxane
Pentostatin
Fludarabine phosphate
Temozolomide
Imiquimod
Carmustine
Clofarabine
Gemcitabine hydrochloride
Exemestane
Celecoxib
Zoledronic acid
Lenalidomide
Nelarabine
Vismodegib
Belinostat
Cabozantinib
Plerixafor

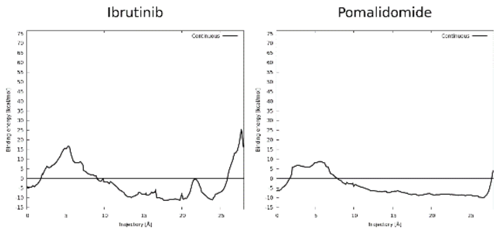

Supplementary Figure S4. Binding energy plots for all successfully finished jobs for the continuous trajectory calculation for the tunnel 3 of cytochrome P450 17A1. Binding energy (in kcal/mol) is plotted against the tunnel length (in Å).

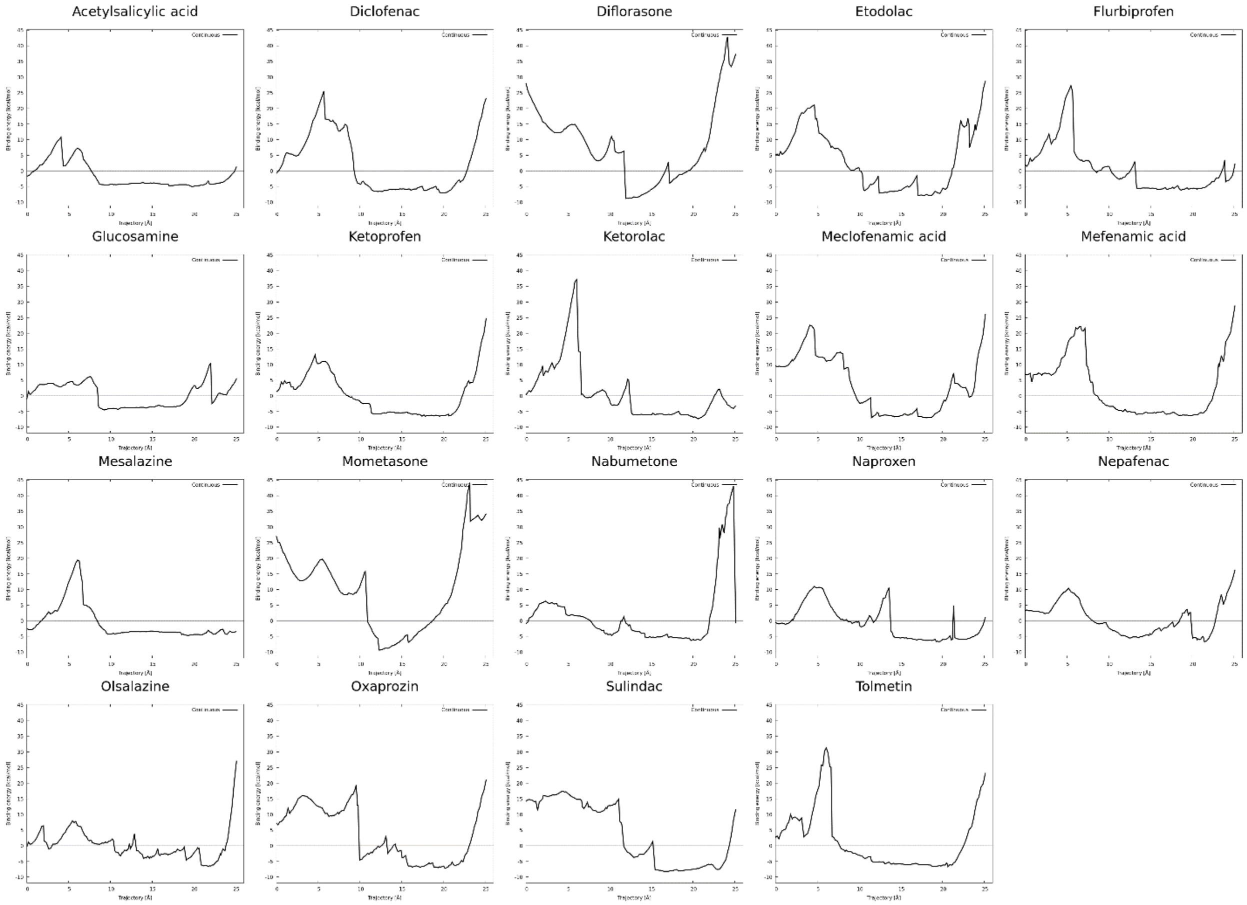

Supplementary Figure S5. Binding energy plots for all successfully finished jobs for the continuous trajectory calculation for the tunnel LTA4 of leukotriene A4 hydrolase/aminopeptidase. Binding energy (in kcal/mol) is plotted against the tunnel length (in Å).

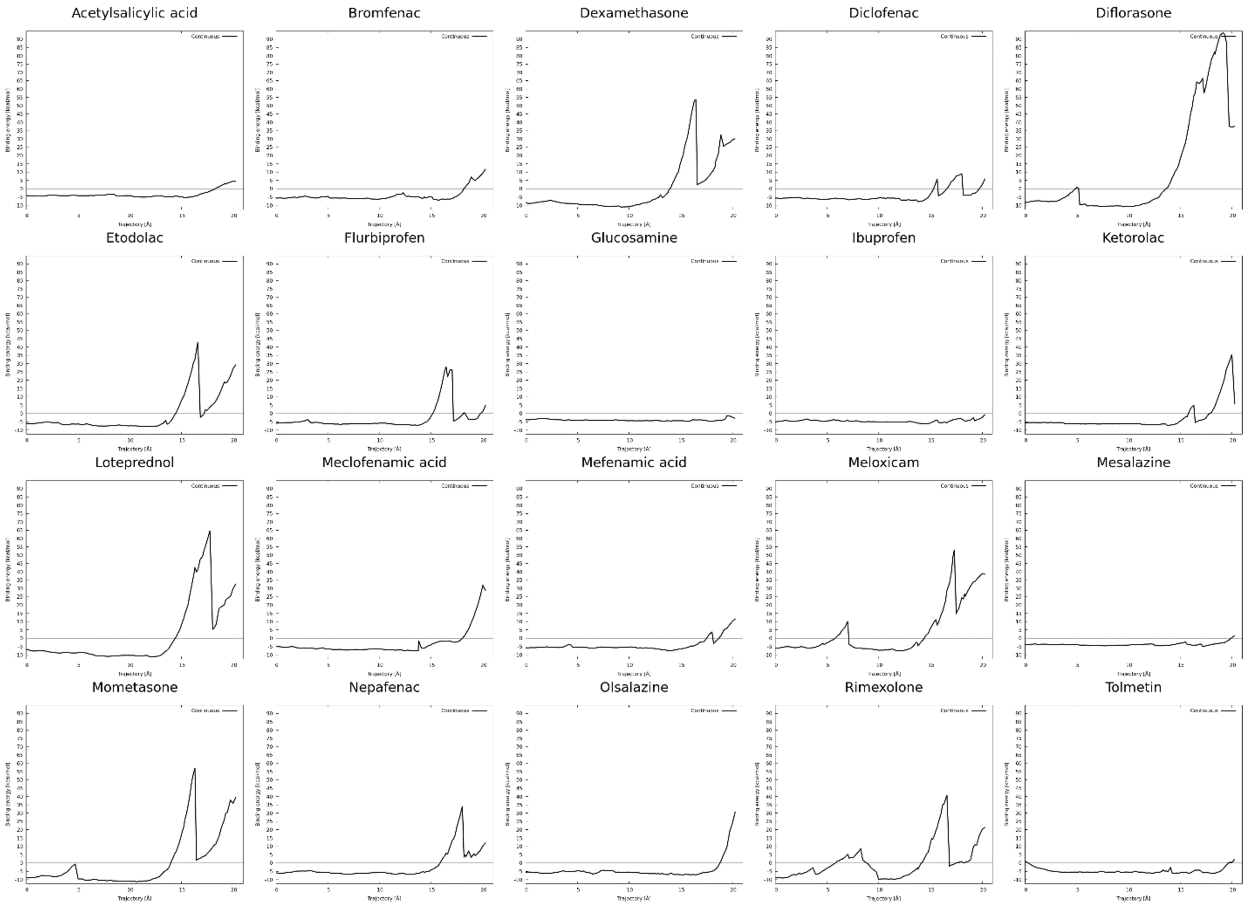

Supplementary Figure S6. Binding energy plots for all successfully finished jobs for the continuous trajectory calculation for the tunnel PGP of leukotriene A4 hydrolase/aminopeptidase. Binding energy (in kcal/mol) is plotted against the tunnel length (in Å).

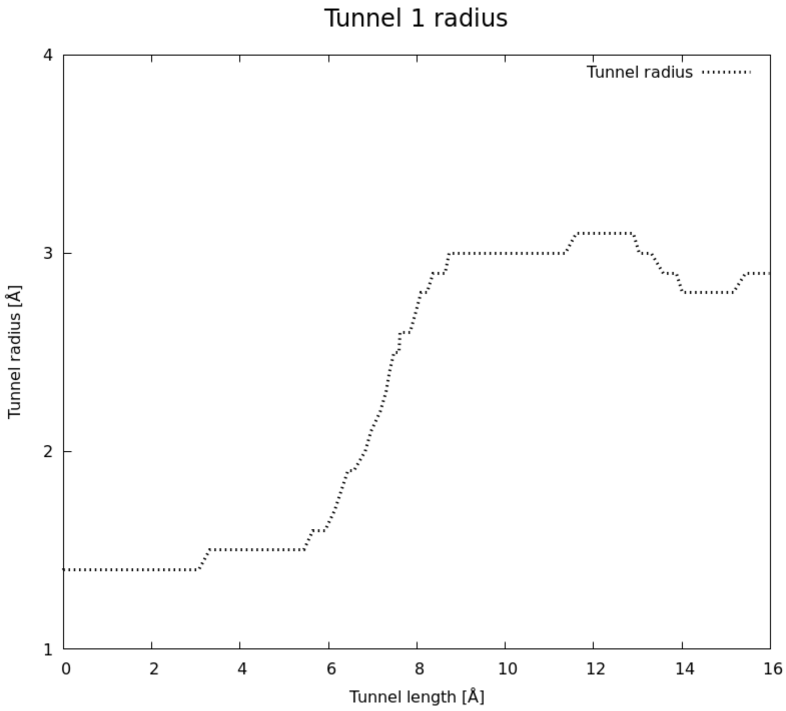

Supplementary Figure S7. Profile of the tunnel 1 of cytochrome P450 17A1.

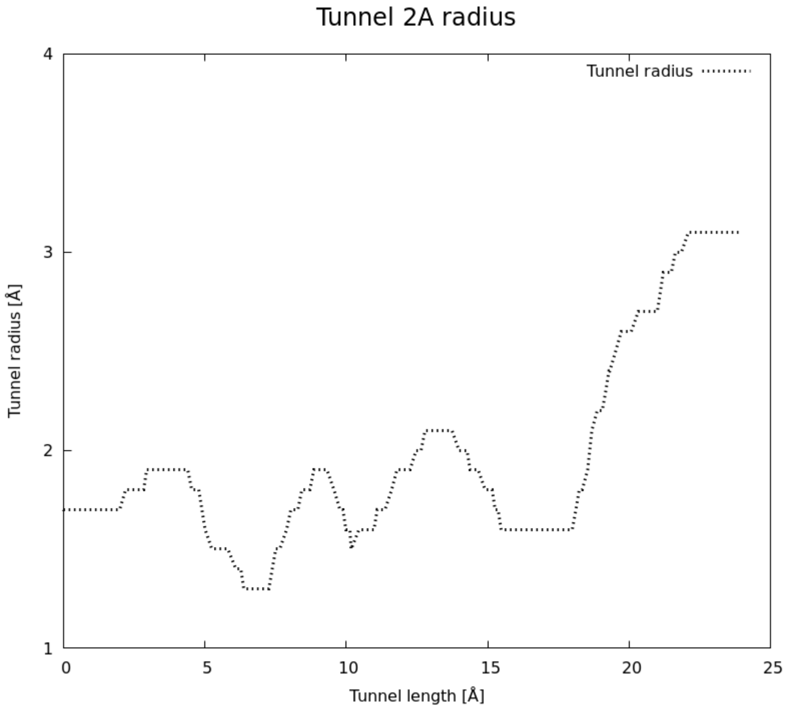

Supplementary Figure S8. Profile of the tunnel 2A of cytochrome P450 17A1.

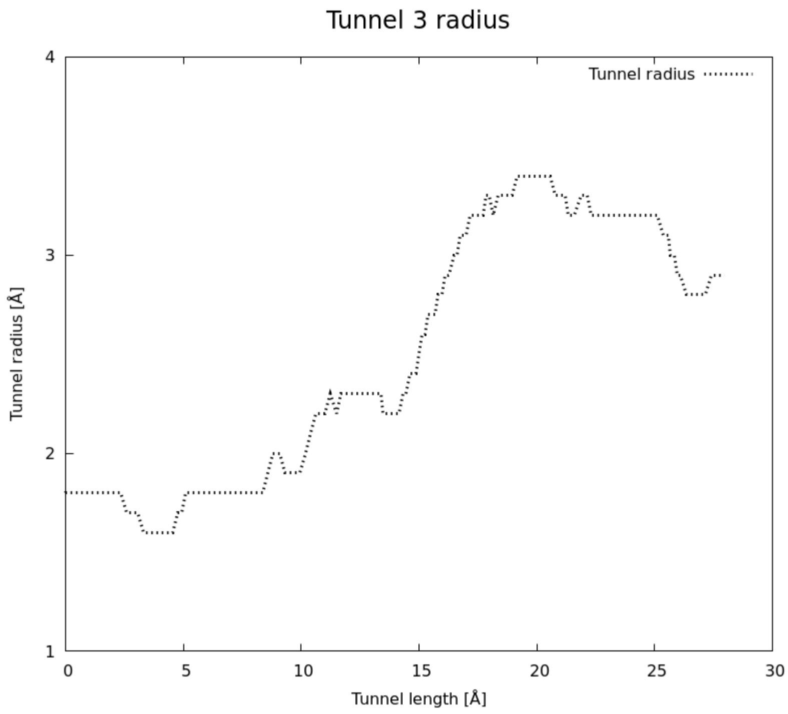

Supplementary Figure S9. Profile of the tunnel 3 of cytochrome P450 17A1.

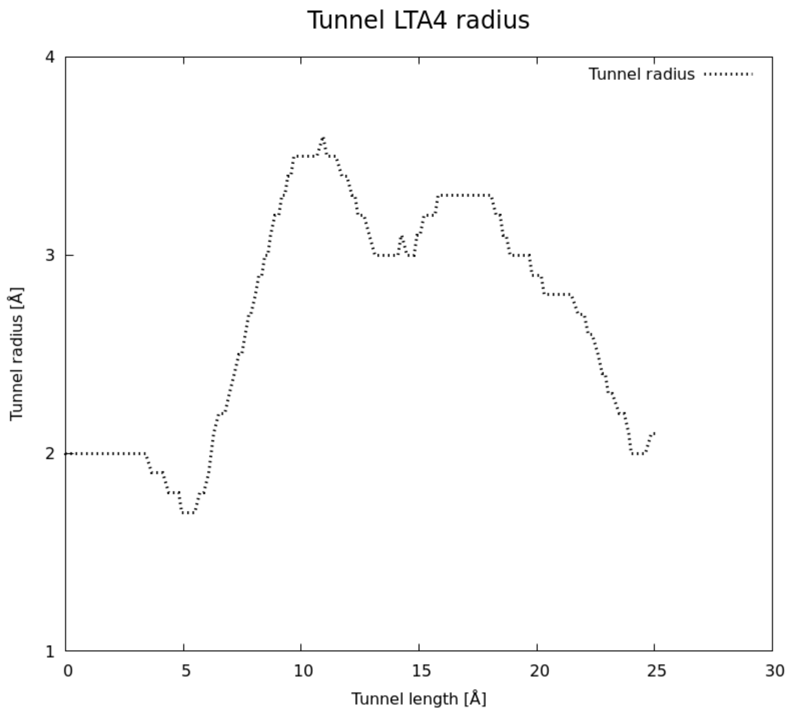

Supplementary Figure S10. Profile of the tunnel LTA4 of leukotriene A4 hydrolase/aminopeptidase.

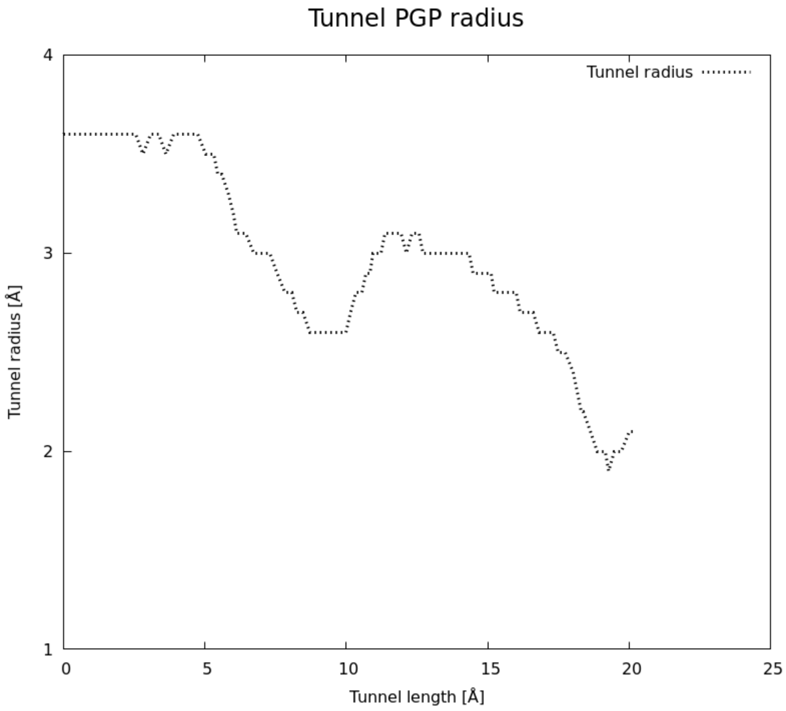

Supplementary Figure S11. Profile of the tunnel PGP of leukotriene A4 hydrolase/aminopeptidase.

Supplementary Table S4. Root mean square deviation (RMSD) of CaverDock *versus* AutoDock Vina minima for the target cytochrome P450 17A1. Lower bound calculations are presented for finished cases.

	**Tunnel 1 CaverDock Lower Bound vs AutoDock Vina - RMSD (Å)**	**Tunnel 2A CaverDock Lower Bound vs AutoDock Vina - RMSD (Å)**	**Tunnel 3 CaverDock Lower Bound vs AutoDock Vina - RMSD (Å)**
Methotrexate	1.941	7.666	1.694
Busulfan	1.704	5.601	1.326
Thioguanine	0.128	0.211	0.21
Mercaptopurine	3.535	0.35	3.501
Mechlorethamine hydrochloride	4.528	0.358	0.301
Allopurinol	1.994	1.89	1.938
Chlorambucil	1.307	7.481	7.644
Thiotepa	0.657	0.691	0.127
Triethylenemelamine	4.543	5.746	0.554
Altretamine	1.361	0.957	0.978
Fluorouracil	0.309	0.263	0.345
Plicamycin	14.788		
Pipobroman	0.318	0.37	0.197
Cyclophosphamide	3.245	2.285	3.237
Mitomycin	6.618	7.023	6.619
Floxuridine	4.61	4.323	2.936
Hydroxyurea	0.293	0.209	0.363
Uracil mustard	5.155	5.588	1.337
Mitotane	5.246	5.247	5.234
Dacarbazine	4.811	4.862	1.265
Methoxsalen	6.453	0.475	0.264
Thalidomide	0.294	3.995	2.235
Megestrol acetate	0.707	7.716	3.134
Trifluridine	0.988	6.989	0.259
Lomustine	1.363	5.868	2.023
Streptozocin	0.33	0.461	0.599
Azacitidine	6.403	3.662	6.367
Cladribine	1.828	4.02	0.257
Ifosfamide	0.874	4.312	4.386
Tretinoin	1.137	3.763	1.159
Teniposide	2.033		
Decitabine	2.885	2.659	2.461
Bendamustine hydrochloride	0.371	8.89	0.764
Etoposide	0.147		0.261
Dexrazoxane	2.928	6.861	2.071
Pentostatin	0.172	3.911	1.569
Sirolimus	9.125		
Valrubicin	0.988		
Idarubicin hydrochloride	1.039		0.652
Mitoxantrone	7.269	9.219	7.511
Amifostine	5.497	5.656	2.855
Fludarabine phosphate	0.299	0.745	0.395
Temozolomide	0.135	0.17	0.12
Imiquimod	0.883	4.8	0.869
Carmustine	0.373	4.555	1.082
Clofarabine	1.714	4.3	0.147
Vorinostat	1.859	7.581	2.696
Capecitabine	3.604	7.388	0.359
Exemestane	0.119	4.075	0.228
Gefitinib	2.813	8.187	1.052
Fulvestrant	2.78	1.315	0.733
Anastrozole	0.897	4.319	0.126
Letrozole	6.482	6.018	6.551
Celecoxib	5.773	6.994	1.326

Zoledronic acid	1.292	1.555	1.654
Dasatinib	2.695	12.302	0.109
Everolimus	8.859		
Imatinib	11.942	7.076	0.061
Lapatinib	1.995	1.989	1.354
Nilotinib	5.858	13.392	0.694
Sorafenib	2.161	12.781	2.793
Lenalidomide	0.166	7.045	0.164
Ixabepilone	1.332		
Raloxifene	1.151	10.282	3.274
Abiraterone	0.11	8.023	0.156
Sunitinib	1.997	11.148	0.225
Afatinib	1.214	6.169	0.21
Olaparib	0.196	10.487	0.383
Pralatrexate	2.496	6.568	2.625
Enzalutamide	1.015	0.951	
Lenvatinib	1.453	4.405	0.267
Nelarabine	0.221	7.178	0.219
Vismodegib	2.325	10.628	2.548
Rucaparib phosphate	0.365	8.854	0.233
Crizotinib	8.337	5.532	0.955
Axitinib	1.778	8.901	9.631
Trametinib		0.196	0.163
Palbociclib	4.22	3.897	0.775
Carfilzomib	2.827		
Omacetaxine mepesuccinate	0.354	4.996	2.739
Ponatinib	7.068	7.157	7.108
Belinostat	2.164	7.887	7.961
Idelalisib	1.036		3.627
Vandetanib	3.918	4.802	0.161
Cabozantinib	6.684	6.746	6.699
Panobinostat	7.015	6.481	7.677
Erismodegib	14.23	14.203	0.122
Plerixafor	1.343	1.455	5.273
Vemurafenib	2.661	11.358	1.176
Ibrutinib	1.196	1.896	0.122
Regorafenib	2.29	2.144	2.266
Alectinib	12.465		
Bosutinib	0.524	0.558	0.362
Venetoclax	3.143		
Cobimetinib	4.448	7.203	1.69
Pomalidomide	0.296	0.307	0.175
Ceritinib	9.207	11.022	2.979
Ribociclib	1.23	10.922	0.177
Osimertinib	0.733	9.723	0.2
Uridine triacetate	1.059	1.984	0.263

Supplementary Table S5. Root mean square deviation (RMSD) of CaverDock *versus* AutoDock Vina minima for the target cytochrome P450 17A1. Upper bound calculations are presented for finished cases.

	Tunnel 1 CaverDock Continuous vs AutoDock Vina - RMSD (Å)	Tunnel 2A CaverDock Continuous vs AutoDock Vina - RMSD (Å)	Tunnel 3 CaverDock Continuous vs AutoDock Vina - RMSD (Å)
Busulfan			15.966
Thioguanine	7.436	8.27	7.85
Mercaptopurine	10.407	10.824	9.379
Mechlorethamine hydrochloride	5.625	10.149	10.394
Allopurinol	8.06	8.608	10.935
Chlorambucil		14.009	
Thiotepa	5.764	10.933	11.377
Triethylenemelamine	7.678	13.884	11.148
Altretamine	7.35	13.669	15.934
Fluorouracil	6.047	7.967	8.694
Pipobroman	6.274	11.625	10.278
Cyclophosphamide	5.105	9.57	
Mitomycin	6.79		
Floxuridine		10.271	7.123
Hydroxyurea	7.456	7.456	20.023
Mitotane	6.234	10.377	9.606
Dacarbazine	6.012	12.422	11.696
Methoxsalen		7.913	7.43
Thalidomide	4.996	11.667	
Megestrol acetate		10.012	
Trifluridine	5.983	13.858	10.785
Lomustine	8.986	10.916	11.011
Azacitidine	5.724	8.549	9.221
Cladribine	7.052	15.263	8.879
Ifosfamide	8.888	9.482	9.506
Decitabine			10.139
Dexrazoxane	8.767	12.094	10.991
Pentostatin	7.848	9.8	7.984
Amifostine	5.633		
Fludarabine phosphate	6.726	11.269	9.117
Temozolomide	5.797	10.645	7.704
Imiquimod	6.977	9.389	11.414
Carmustine	6.717	8.584	10.643
Clofarabine	7.258	12.582	7.335
Exemestane	6.232	10.622	8.000
Anastrozole	8.115		
Celecoxib			13.645
Zoledronic acid	6.488	8.133	7.748
Lenalidomide	4.943	11.07	8.968
Abiraterone		11.691	
Enzalutamide	5.478		
Nelarabine	8.067	13.751	12.702
Vismodegib			13.5
Rucaparib phosphate	7.025	10.95	
Axitinib		13.122	
Ponatinib	6.181		
Belinostat	6.605		12.436
Cabozantinib	7.616		13.034
Plerixafor			7.394
Vemurafenib		15.39	
Ibrutinib			10.689
Bosutinib	5.041		
Cobimetinib	8.923		
Pomalidomide	6.132	7.166	9.037

Supplementary Table S6. Root mean square deviation (RMSD) of CaverDock *versus* AutoDock Vina minima for the target leukotriene A4 hydrolase/aminopeptidase. Lower bound calculations are presented for finished cases.

	Tunnel LTA4 CaverDock Lower Bound vs AutoDock Vina - RMSD (Å)	Tunnel PGP CaverDock Lower Bound vs AutoDock Vina - RMSD (Å)
Acetylsalicylic_acid	3.815	0.514
Balsalazide	7.686	1.656
Beclomethasone_dipropionate		3.242
Betamethasone	8.434	0.064
Bromfenac	5.276	5.322
Clobetasol_propionate	2.072	1.706
Clocortolone	7.11	3.641
Cromoglicic_acid		10.255
Desoximetasone	7.576	0.187
Dexamethasone	8.426	0.121
Diclofenac	0.131	0.862
Difenoxin	9.517	0.366
Diflorasone	7.398	0.341
Diphenoxylate	9.654	1.743
Etodolac	0.274	6.545
Fenoprofen	1.97	1.548
Flunisolide	0.431	0.08
Flurbiprofen	1.311	1.287
Fluticasone_propionate	10.36	0.176
Glucosamine	12.324	12.313
Hydrocortisone	3.951	0.144
Ibuprofen	5.124	4.602
Indomethacin	10.685	7.577
Ketoprofen	7.059	6.906
Ketorolac	3.378	1.524
Loperamide	7.482	0.102
Loteprednol	6.899	0.123
Meclofenamic_acid	0.329	0.385
Mefenamic_acid	2.567	0.899
Meloxicam	10.962	0.275
Mesalazine	12.518	12.686
Methylprednisolone	7.89	0.287
Mometasone	7.829	0.366
Nabumetone	2.442	0.795
Naproxen	2.051	0.341
Nepafenac	0.97	4.387
Olsalazine	1.197	0.402
Oxaprozin	4.098	1.447
Paromomycin	0.842	7.014
Piroxicam	9.998	7.791
Prednisolone	4.25	0.285
Prednisone	2.816	8.524
Rabeprazole	5.038	8.407
Rimexolone	8.275	0.195
Roflumilast	6.908	9.000
Streptomycin	8.427	4.286
Sulindac	1.067	0.222
Tolmetin	1.853	0.236
Triamcinolone	8.362	0.171

Supplementary Table S7. Root mean square deviation (RMSD) of CaverDock minima *versus* AutoDock Vina minima for the target leukotriene A4 hydrolase/aminopeptidase. Continuous trajectory calculations are presented for finished cases.

	Tunnel LTA4 CaverDock Continuous vs AutoDock Vina - RMSD (Å)	Tunnel PGP CaverDock Continuous vs AutoDock Vina - RMSD (Å)
Acetylsalicylic_acid	9.965	6.865
Bromfenac		7.139
Dexamethasone		3.597
Diclofenac	11.544	8.428
Diflorasone	10.204	5.936
Etodolac	11.907	8.783
Flurbiprofen	11.544	9.081
Glucosamine	8.186	12.961
Ibuprofen		9.268
Ketoprofen	11.589	
Ketorolac	13.555	5.567
Loteprednol		2.511
Meclofenamic acid	10.942	6.428
Mefenamic acid	12.138	7.500
Meloxicam		6.895
Mesalazine	21.069	12.563
Mometasone	9.763	5.146
Nabumetone	11.041	
Naproxen	12.523	
Nepafenac	10.94	10.483
Olsalazine	11.183	3.966
Oxaprozin	10.93	
Rimexolone		6.266

References

Arolas, J. L., Botelho, T. O., Vilcinskas, A., and Gomis-Rüth, F. X. (2011). Structural evidence for standard-mechanism inhibition in metallopeptidases from a complex poised to resynthesize a peptide bond. *Angew. Chem. Int. Ed.* 50, 10357–10360. doi:10.1002/anie.201103262.

Čolović, M. B., Krstić, D. Z., Lazarević-Pašti, T. D., Bondžić, A. M., and Vasić, V. M. (2013). Acetylcholinesterase inhibitors: pharmacology and toxicology. *Curr. Neuropharmacol.* 11, 315–335. doi:10.2174/1570159X11311030006.

Silman, I., and Sussman, J. L. (2005). Acetylcholinesterase: “classical” and “non-classical” functions and pharmacology. *Curr. Opin. Pharmacol.* 5, 293–302. doi:10.1016/j.coph.2005.01.014.

Geller, D. A., and Billiar, T. R. (1998). Molecular biology of nitric oxide synthases. *Cancer Metastasis Rev.* 17, 7–23.

Cannon, C. P. (2011). High-density lipoprotein cholesterol as the Holy Grail. *JAMA* 306, 2153–2155. doi:10.1001/jama.2011.1687.

Dullaart, R. P. F., Dallinga-Thie, G. M., Wolffenbuttel, B. H. R., and van Tol, A. (2007). CETP inhibition in cardiovascular risk management: a critical appraisal. *Eur. J. Clin. Invest.* 37, 90–98. doi:10.1111/j.1365-2362.2007.01756.x.

Joy, T., and Hegele, R. A. (2008). Is raising HDL a futile strategy for atheroprotection? *Nat. Rev. Drug Discov.* 7, 143–155. doi:10.1038/nrd2489.

Liu, S., Mistry, A., Reynolds, J. M., Lloyd, D. B., Griffor, M. C., Perry, D. A., et al. (2012). Crystal structures of cholesteryl ester transfer protein in complex with inhibitors. *J. Biol. Chem.* 287, 37321–37329. doi:10.1074/jbc.M112.380063.

Bian, K., and Murad, F. (2003). Nitric oxide (NO) - biogeneration, regulation, and relevance to human diseases. *Front. Biosci.* 8, d264-278.

Duncan, A. J., and Heales, S. J. R. (2005). Nitric oxide and neurological disorders. *Mol. Aspects Med.* 26, 67–96. doi:10.1016/j.mam.2004.09.004.

Nathan, C. (2004). The moving frontier in nitric oxide-dependent signaling. *Sci. STKE* 2004, pe52. doi:10.1126/stke.2572004pe52.

Garcin, E. D., Arvai, A. S., Rosenfeld, R. J., Kroeger, M. D., Crane, B. R., Andersson, G., et al. (2008). Anchored plasticity opens doors for selective inhibitor design in nitric oxide synthase. *Nat. Chem. Biol.* 4, 700–707. doi:10.1038/nchembio.115.

Chemonges, S. (2014). The recognition of LpxC inhibitors as potential antibiotics could revolutionise the management of sepsis in veterinary patients if their unknown biological properties are widely evaluated in suitable animal models. *Int. J. Vet. Sci. Med.* 2, 99–102. doi:10.1016/j.ijvsm.2014.10.003.

Tomaras, A. P., McPherson, C. J., Kuhn, M., Carifa, A., Mullins, L., George, D., et al. (2014). LpxC inhibitors as new antibacterial agents and tools for studying regulation of lipid A biosynthesis in Gram-negative pathogens. *MBio* 5, e01551-01514. doi:10.1128/mBio.01551-14.

Whittington, D. A., Rusche, K. M., Shin, H., Fierke, C. A., and Christianson, D. W. (2003). Crystal structure of LpxC, a zinc-dependent deacetylase essential for endotoxin biosynthesis. *Proc. Natl. Acad. Sci. U.S.A.* 100, 8146–8150. doi:10.1073/pnas.1432990100.

Cole, K. E., Gattis, S. G., Angell, H. D., Fierke, C. A., and Christianson, D. W. (2011). Structure of the metal-dependent deacetylase LpxC from Yersinia enterocolitica complexed with the potent inhibitor CHIR-090. *Biochemistry* 50, 258–265. doi:10.1021/bi101622a.

Stöcker, W., and Bode, W. (1995). Structural features of a superfamily of zinc-endopeptidases: the metzincins. *Curr. Opin. Struct. Biol.* 5, 383–390.

Wasserman, Z. R. (2005). Making a new turn in matrix metalloprotease inhibition. *Chem. Biol.* 12, 143–144. doi:10.1016/j.chembiol.2005.01.008.

Engel, C. K., Pirard, B., Schimanski, S., Kirsch, R., Habermann, J., Klingler, O., et al. (2005). Structural basis for the highly selective inhibition of MMP-13. *Chem. Biol.* 12, 181–189. doi:10.1016/j.chembiol.2004.11.014.
