# Supplementary material for: Fast Screening of Inhibitor Binding/Unbinding Using Novel Software Tool CaverDock
Source: Front Chem. 2019 Oct 29;7:709. doi: 10.3389/fchem.2019.00709 (PMC6828983; doi:10.3389/fchem.2019.00709)
Supplement: Supplementary file 2 [file Data_Sheet_2.PDF]

Exhaustiveness = 30

Inhibitor	E_bound	E_max	E_surface	k_barier	δE_bs
Acetylsalicylic_acid	-5.6	0.4	0	6	-5.6
Clocortolone	-10.5	23.5	0	34	-10.5
Cromoglicic_acid	-10.7	56.7	0	67.4	-10.7
Desoximetasone	-10.3	23.9	0	34.2	-10.3
Dexamethasone	-11.1	27.3	0	38.4	-11.1
Diclofenac	-7.9	4.8	0	12.7	-7.9
Difenoxin	-12	14.9	0	26.9	-12
Balsalazide	-8	-5.8	-6	2.2	-2
Betamethasone	-11.1	27.4	0	38.5	-11.1
Bromfenac	-7.5	-1.8	-1.8	5.7	-5.7

Exhaustiveness = 1

Inhibitor	E_bound	E_max	E_surface	k_barier	δE_bs
Acetylsalicylic_acid	-5.5	0.2	0	5.7	-5.5
Clocortolone	-10.4	24.5	0	34.9	-10.4
Cromoglicic_acid	-10.6	57.8	0	68.4	-10.6
Desoximetasone	-10.3	24.5	0	34.8	-10.3
Dexamethasone	-11.1	27.5	0	38.6	-11.1
Diclofenac	-7.8	5.2	0	13	-7.8
Difenoxin	-11.6	15.3	0	26.9	-11.6
Balsalazide	-7.9	-5.3	-5.7	2.6	-2.2
Betamethasone	-11.1	27.9	0	39	-11.1
Bromfenac	-7.5	-0.4	-1.7	7.1	-5.8

	**ΔE(High Exhaustiveness - Low Exhaustiveness)**				
Inhibitor	*ΔE_bound*	*ΔE_max*	*ΔE_surface*	*Δk_barier*	*ΔδE_bs*
Acetylsalicylic_acid	-0.1	0.2	0	0.3	-0.1
Clocortolone	-0.1	-1	0	-0.9	-0.1
Cromoglicic_acid	-0.1	-1.1	0	-1	-0.1
Desoximetasone	0	-0.6	0	-0.6	0
Dexamethasone	0	-0.2	0	-0.2	0
Diclofenac	-0.1	-0.4	0	-0.3	-0.1
Difenoxin	-0.4	-0.4	0	0	-0.4
Balsalazide	-0.1	-0.5	-0.3	-0.4	0.2
Betamethasone	0	-0.5	0	-0.5	0
Bromfenac	0	-1.4	-0.1	-1.4	0.1
